# Cotinine and 6-Hydroxy-L-Nicotine Reverses Memory Deficits and Reduces Oxidative Stress in Aβ_25-35_-Induced Rat Model of Alzheimer’s Disease

**DOI:** 10.3390/antiox9080768

**Published:** 2020-08-18

**Authors:** Razvan Stefan Boiangiu, Marius Mihasan, Dragos Lucian Gorgan, Bogdan Alexandru Stache, Brindusa Alina Petre, Lucian Hritcu

**Affiliations:** 1 Department of Biology, Faculty of Biology, Alexandru Ioan Cuza University of Iasi, 700506 Iasi, Romania; razvan.boiangiu@student.uaic.ro (R.S.B.); marius.mihasan@uaic.ro (M.M.); lucian.gorgan@uaic.ro (D.L.G.); alexandru.stache@student.uaic.ro (B.A.S.); 2 Center for Fundamental Research and Experimental Development in Translation Medicine—TRANSCEND, Regional Institute of Oncology, 700483 Iasi, Romania; brindusa.petre@uaic.ro; 3 Department of Chemistry, Faculty of Chemistry, Alexandru Ioan Cuza University of Iasi, 700506 Iasi, Romania

**Keywords:** Alzheimer’s disease, nicotine, cotinine, 6-hydroxy-L-nicotine, Aβ_25-35_ peptide, memory, oxidative stress, nicotinic acetylcholine receptors, neuroprotection, inflammation

## Abstract

The nicotinic derivatives, cotinine (COT), and 6-hydroxy-L-nicotine (6HLN), showed promising cognitive-improving effects without exhibiting the nicotine’s side-effects. Here, we investigated the impact of COT and 6HLN on memory impairment and the oxidative stress in the Aβ_25-35_-induced rat model of Alzheimer’s disease (AD). COT and 6HLN were chronically administered to Aβ_25-35_-treated rats, and their memory performances were assessed using in vivo tasks (Y-maze, novel object recognition, and radial arm maze). By using in silico tools, we attempted to associate the behavioral outcomes with the calculated binding potential of these nicotinic compounds in the allosteric sites of α7 and α4β2 subtypes of the nicotinic acetylcholine receptors (nAChRs). The oxidative status and acetylcholinesterase (AChE) activity were determined from the hippocampal tissues. RT-qPCR assessed *bdnf, arc, and il-1β mRNA* levels. Our data revealed that COT and 6HLN could bind to α7 and α4β2 nAChRs with similar or even higher affinity than nicotine. Consequently, the treatment exhibited a pro-cognitive, antioxidant, and anti-AChE profile in the Aβ_25-35_-induced rat model of AD. Finally, RT-qPCR analysis revealed that COT and 6HLN positively modulated the *bdnf*, *arc,* and *il-1β* genes expression. Therefore, these nicotinic derivatives that act on the cholinergic system might represent a promising choice to ameliorate AD conditions.

## 1. Introduction

Alzheimer’s disease (AD) is a progressive, unremitting neurodegenerative disease and represents the most typical cause of dementia in the elderly population. With approximately 30–35 million people affected worldwide and, according to the World Health Organization (WHO), this number is predicted to triple due to life expectancy on the rise [1,2]. AD manifests by a severe and gradual memory loss, which is accompanied by several pathologic hallmarks such as the formation of extra- and intracellular senile plaques of beta-amyloid (Aβ), the intraneuronal formation of neurofibrillary tangles (NFTs) of hyperphosphorylated tau protein and death of cholinergic neurons in the hippocampus with a reduction in acetylcholine (ACh) levels [3,4,5]. Even though the etiological agent is unknown, in recent decades, several hypotheses regarding AD pathogenesis emerged. Amyloid cascade hypothesis [6] postulates that neurodegeneration from AD is provoked by an abnormal accumulation of senile plaques of Aβ in different areas of the brain, which acts as a pathological trigger for a cascade including synaptic failure, neuronal dysfunction and cell death [7]. At present, it is generally considered that an imbalance between Aβ production and clearance is an initiating factor in AD [8]. The cholinergic hypothesis states that the cognitive decline observed in AD is caused by the loss of cholinergic function in the central nervous system (CNS) [9]. It is considered that the deterioration of cognitive function observed in AD patients is caused by the degeneration of the cholinergic neurons in the basal forebrain and associated loss of cholinergic neurotransmission in the cerebral cortex and hippocampus [10].

Nicotinic acetylcholine receptors (nAChRs) represent the main target of ACh in the brain. Their presence in cholinergic neurons indicates involvement in higher cognitive functions, including memory and learning [11,12]. nAChRs are transmembrane pentameric ligand-gated ion channels composed by combining different α and β subunits, which confer the pharmacological and kinetic properties of the receptor. In the human nervous system, eight different α (α2–α7, α9, α10) and three different β (β2–β4) subunits exist. By assembling these subunits in different combinations, a wide range of nAChRs subtypes with distinct electrophysiological properties and brain localization are generated [12]. Considering that α7 and α4β2 subtypes of nAChRs are involved in AD pathogenesis [13], the use of nAChRs modulators to increase the availability for ACh and to overcome the death of the forebrain cholinergic neurons represent a new therapeutic strategy for AD [14].

Nicotine (NIC) is an alkaloid extracted from *Nicotiana tabacum* and an exogenous agonist of nAChRs [15]. Accumulated evidence from animal and clinical studies showed that NIC has cognitive-enhancing effects, improving performance in several domains of cognition, including attention, learning, and working memory [16]. Moreover, NIC acts as an antioxidant, reducing the oxidative stress [17], has anti-inflammatory effects [18], and is neuroprotective against Aβ, reducing its aggregation in the brain [19]. However, the cardiovascular [20] and addictive [21] side effects of NIC along with negative publicity related to smoking [22] have limited its therapeutic use in AD.

In humans, about 80% of NIC is metabolized by the liver cytochrome P450 2A6 (CYP2A6) to cotinine (COT, (5S)-1-methyl-5-(pyridin-3-yl) pyrrolidin-2-one). (S)-Cotinine, the physiologically active isomer of COT, accumulates in the body as a result of tobacco exposure [23]. COT is 100-fold less toxic than NIC, has a longer half-life (20–24 vs. 2 h, respectively), is capable of crossing the blood–brain barrier, and does not cause addiction [23]. This difference between the half-life of COT and NIC in the blood supports the idea that COT could underlie the prolonged effects of NIC in CNS [24].

6-Hydroxy-L-nicotine (6HLN, 5-[(2S)-1-methylpyrrolidin-2-yl]pyridin-2-ol) is the first metabolic intermediate found in NIC catabolic pathway encoded by pAO1 megaplasmid of *Paenarthrobacter nicotinovorans* [25]. 6HLN results from the hydroxylation reaction of L-nicotine catalyzed by nicotine-dehydrogenase (NDH, EC 1.5.99.4), a trimer enzyme encoded by the *ndhL*, *ndhM,* and *ndhS* genes of pAO1 [26]. During NIC metabolization, 6HLN is temporarily accumulated in the media [27] and is further dehydrogenated in 6-hydroxy-methylmyosmine by 6-hydroxy-L-nicotine oxidase (6HLNO, EC 1.5.3.5).

Previously, our group demonstrated that 6HLN improve spatial memory in the behavioral tasks and enhanced oxidative status in the hippocampus of the chlorisondamine (CHL)-treated rats [28]. Moreover, several other laboratories showed that COT reduced Aβ deposition and ameliorated cognitive impairment in AD mice [29,30]. Therefore, this study aimed to investigate the binding potential of COT and 6HLN in different subtypes of nAChRs and to evaluate the effects of chronic intraperitoneal (i.p.) administration of these compounds on memory deficits and oxidative stress in a rat model of AD induced by intracerebroventricular (i.c.v.) infusion of Aβ_25-35_ peptide. Additionally, we examined the effect of the treatment on the hippocampal *bdnf*, *arc,* and *il-1β* mRNA expression.

## 2. Materials and Methods

### 2.1. Molecular Docking Simulations

#### 2.1.1. Tridimensional (3D) Structures of Ligands and Receptors

For the in silico molecular docking experiments, we used the 3D structures of the acetylcholine binding protein (AChBP) and human α4β2 nAChRs (3α:2β) as receptors. The 3D coordinates were downloaded from Protein Data Bank (PDB) (Table 1) and edited using UCSF Chimera v1.13.1 software (RBVI, CA, USA) [31]. Only the subunits (interface) that formed the NIC binding sites (AChBP, α4-α4 and α4-β2) were retained and used. The co-crystallized native NIC molecule was also removed from the binding sites.

The 3D structures of the ligands were downloaded from PubChem database ((*S*)-Nicotine—89,594, (*R*)-Nicotine—157672, (*S*)-Cotinine—854,019, (*R*)-Cotinine—21,907, (*S*)-6-Hydroxynicotine—439,383 and (*R*)-6-Hydroxynicotine—439,886) and further converted into a proper format for molecular docking using Frog v2.14—free online drug conformation generation (RPBS, Paris, France) [34]. Our study focused on the (*S*) enantiomers of the three ligands because (*S*)-nicotine is the predominant form in nature, and the resulting derivatives from its degradation are (*S*)-cotinine and (*S*)-6-hydroxynicotine, respectively. However, in our docking simulations, we also included the (*R*) enantiomers of the selected ligands. Ligplot^+^ v2.2 (EMBL-EBI, Cambridge, United Kingdom) [35] was used to generate bidimensional (2D) diagrams of ligand-receptor interactions that were subsequently visualized in 3D with PyMOL v2.2.3 (Schrӧdinger, NY, USA) [36].

#### 2.1.2. Docking Software and Parameters

In silico docking was performed using the genetic algorithm provided by AutoDock 4 [37]. All the steps required for docking (the generation of the molecular surface, energy grid, and search box) as well as the evaluation of the docking results, were accomplished using AutoDockTools v1.5.6 (ADT, Scripps Research, La Jolla, CA, USA) [38] with default parameters. The receptors were kept rigid, and all ligands were flexible. All bonds within ligand molecules could rotate. The docking was local, and the search area was a 60 × 60 × 60 Å box centered on the NIC binding site. The resulting conformations were overlapped over the original 3D structure of the native ligand from PDB, and the Root Mean Square Deviation (RMSD) was calculated using PdbViewer v4.1.0 (SIB, Lausanne, Switzerland) [39].

### 2.2. Animals

A total number of 50 male Wistar rats, age 4–5-months and an average weight of 350 g (±10 g) at the beginning of the experiment, were purchased from Cantacuzino Institute (Bucharest, Romania). The rodents were housed in a room with light (12 h light/dark cycle, starting at 8:00 a.m.) and temperature (22 °C) conditions controlled and with access to food and water *ad libitum*. Efforts were made to minimize the number of animals used in the experiment and their potential suffering. The experiments involving animal use were performed according to the European Communities Council Directive (Directive 2010/63/EU) regarding the protection of animals used for scientific purposes with approval of the Ethical Committee (No. 15309/22.07.2019).

### 2.3. Neurosurgery

**Figure 1:**
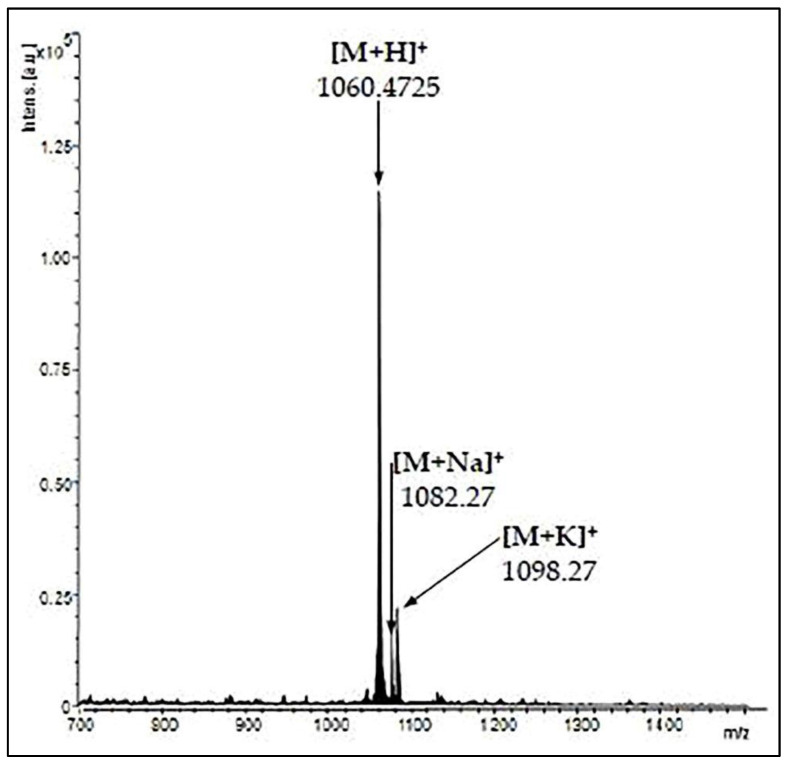
Matrix Assisted Laser Desorption/Ionization—Time of Flight (MALDI-TOF) mass spectrum of Aβ_25–35_ peptide.

**Figure 2:**
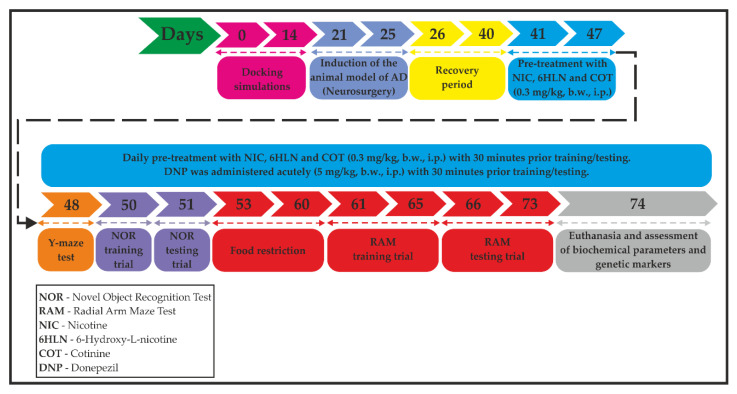
A flowchart showing the timeline and experimental design.

The animal model of AD was induced using the stereotaxic procedure performed in aseptic conditions, as previously described by Postu et al. [40] and Fedotova et al. [41]. Briefly, the rats were anesthetized using sodium pentobarbital salt (50 mg/kg, b.w., i.p., Sigma-Aldrich, Darmstadt, Germany) and were placed in the stereotaxic apparatus with the nose orientated 11° below horizontal zero plane. An incision was made on the midline of the scalp and a hole was drilled in the skull according to the following coordinates [42]: 1.5 mm lateral to the midline, 7.4 mm ventral to the surface of the cortex and 0.0 mm anteroposterior. A total volume of 4 µL of Aβ_25-35_ peptide solution (0.5 mg/mL) was right-unilaterally and gradually (1 µL/min) injected using a Hamilton syringe. The Aβ_25-35_ peptide was manually synthesized through Solid-Phase Peptide Synthesis (SPPS) and characterized by mass spectrometry (Figure 1) and was kindly provided by Brindusa Alina Petre, Ph.D. (Department of Chemistry, Alexandru Ioan Cuza University of Iasi, Romania). The peptide solution was prepared by dissolving Aβ_25-35_ in sterile saline solution (0.9% NaCl), and the mixture was incubated for one week at 37 °C to allow the peptide to form aggregates (fibrils) [43]. In the control group, the Aβ_25-35_ solution was replaced with the same volume of vehicle. Post-surgery, special care was given to the rats until the spontaneous feeding was restored, and two weeks after surgery, the behavioral tasks were conducted (Figure 2).

### 2.4. Drug Treatment and Group Division

NIC (CAS no. 54-11-5), COT (CAS no. 486-56-6), and donepezil (DNP, CAS no. 120011-70-3) of high purity were purchased from Sigma-Aldrich, Darmstadt, Germany. 6HLN was obtained by chemical synthesis and was a kind gift from Prof. dr. Roderich Brandsch (Albert-Ludwings University of Freiburg, Freiburg, Germany). Stock solutions of 0.3 mg/mL of NIC, 6HLN, and COT and 5 mg/mL of DNP were prepared freshly by dissolving the compounds in sterile saline solution (0.9% NaCl). The animals were randomly assigned to six experimental groups (six animals per group) as follows: (1) the control group (sham-operated) that received the vehicle; (2) the Aβ_25-35_-treated group that received the vehicle treatment, as negative control; (3) the Aβ_25-35_-treated group that received DNP solution, as a positive control; (4) the Aβ_25-35_-treated group that received NIC solution; (5) the Aβ_25-35_-treated group that received 6HLN solution and (6) the Aβ_25-35_-treated group that received COT solution. We also confirmed that *n* = 6 animals/group is appropriate using InVivoStat, and R-based statistical package [44]. Based on a significance level of 0.05, the power to detect a 20% biologically relevant change from control is 100%. Chronic treatment with NIC, 6HLN, and COT (0.3 mg/kg, b.w., i.p.) began immediately after the recovery period, with one week before and during the behavioral tests, with 30 min prior training or testing, and lasted until euthanasia (33 days). DNP was administered acutely by i.p. injection in a single dose of 5 mg/kg, b.w., with 30 min before training or testing (Figure 2).

### 2.5. Behavior Assessment

#### 2.5.1. Y-Maze Task

Short-term spatial recognition memory and locomotor activity were assessed using a single-session Y-maze test, which was previously described by Jackson [45] and Boiangiu et al. [46]. The apparatus was made from black Plexiglas and consisted of three identical arms with a size of 35 × 10 × 25 cm (L × l × h) joined in a central area in the form of an equilateral triangle shape. Thirty minutes after the drug administration, the rats were individually placed at the end of one arm, and their behavior was recorded for 8 min. A valid entry was taken into consideration only when all paws were inside the arm. The rat’s behavior was recorded using a Logitech HD Webcam C922 Pro Stream camera (Logitech, Lausanne, Switzerland), and the video recordings were analyzed using ANY-maze^®^ software (Stoelting Co., Wood Dale, IL, USA). Locomotor activity was calculated by quantifying the total number of arm entries. Spontaneous alternation behavior is defined by a successive entry in each arm and was calculated in percentages as (number of alternations/total number of arms entries—2) × 100. The floor and the walls of the apparatus were cleaned with 10% ethanol solution between animals.

#### 2.5.2. Novel Object Recognition Task

The novel object recognition (NOR) protocol uses the natural preference of the rodents for novel objects and is used to evaluate the cognitive modifications in rodent models of neurodegenerative disorders [47]. The rodents were trained and tested according to the procedure described previously by Foyet et al. [48] and Boiangiu et al. [46]. The test apparatus consisted of a black square-shaped box made from wood of the size 72 × 72 × 36 cm (L × l × h). The NOR test was divided into three sessions of 5 min each: (1) the habituation session (T_0_) in which the animals were individually placed in the empty apparatus, (2) the training session (T_1_), conducted 24 h later, where the animals were allowed to explore two identical objects placed in opposite corners of the apparatus and (3) the testing session (T_2_), conducted after a 4 h inter-trial period, where one of the familiar objects (F) was replaced with a novel object (N). The entire surface of the apparatus was cleaned with ethanol solution (10%) between animals. The exploratory behavior was taken into consideration when the objects were sniffed or touched with nose or paws and was recorded using a Logitech HD Webcam C922 Pro Stream camera and analyzed using ANY-maze^®^ software (Stoelting Co., Wood Dale, IL, USA). The ability of the animals to discriminate F and N was measured by comparing the exploring time of F (T_F_) and N (T_N_) in T_2_. For this, we determined the discrimination (DI) and recognition (RI) indices. DI was calculated as (T_N_ − T_F_)/(T_N_ + T_F_), whereas RI was calculated as T_N_/(T_N_ + T_F_). DI can vary from +1 to −1, where a positive score suggests more time exploring N, a negative score indicates more time exploring F and a zero score indicates a null preference [49].

#### 2.5.3. Radial Arm Maze Task

The spatial learning and memory of the rats were evaluated using a protocol of the radial arm maze (RAM) task described for the first time by Olton and Samuelson [50] and modified by Hritcu et al. [51]. Our RAM was built from grey-translucent Plexiglass and consisted of 8 arms in size of 40 × 10 × 20 cm (L × l × h, numbered from 1 to 8) radially arranged around a circular arena with 20 cm in diameter. The apparatus was raised 50 cm above the floor. A food restriction period of 7 days was needed to reduce the weight of the animals with 15%. The test was divided into two sessions as follows: (1) the training session (5 min/4 consecutive days) in which the food was initially available throughout the maze and gradually reduced to the food cup located the end of the arms and (2) the testing session (7 consecutive days) in which a food pellet (50 mg) was placed in the cup at the end of arms 1, 2, 4, 5 and 7. In the testing session, the rats were individually placed in the center of the maze and they had up to 5 min to enter the baited arms and to consume the food. An entry in an unbaited arm was considered a reference memory error, whereas an entry in a baited arm that was previously visited was considered a working memory error. The behavior was recorded with a Logitech HD Webcam C922 Pro Stream camera (Logitech, Lausanne, Switzerland) and the video recordings were analyzed using ANY-maze^®^ software (Stoelting, Co., Wood Dale, IL, USA). The maze was cleaned with 10% ethanol solution between sessions.

### 2.6. Biochemical Parameters Assessment

After completing the behavioral tests, the rats were euthanized with an overdose of pentobarbital sodium salt (150 mg/kg, b.w., i.p.) and beheaded. The hippocampi were precisely excised and homogenized on ice in 0.1 M potassium phosphate buffer (pH 7.4) with 1.15% KCl using a Potter Homogenizer (Heidolph Instruments, Schwabach, Germany) coupled with a Servodyne Mixer Controller (Cole-Parmer Instrument Co., Chicago, IL, USA). The homogenates were centrifuged (15 min at 960× *g*) and the supernatant was used to determine superoxide dismutase (SOD), catalase (CAT), glutathione peroxidase (GPX) and acetylcholinesterase (AChE) specific activities and the total content of reduced glutathione (GSH), malondialdehyde (MDA) and carbonylated proteins.

#### 2.6.1. Protein Concentration Estimation

Soluble protein concentration was determined using the Bicinchoninic Acid (BCA) Kit (Sigma-Aldrich, Darmstadt, Germany) that is based on the principle of Smith et al. [52].

#### 2.6.2. AChE Activity Determination

AChE (E.C. 3.1.1.7) activity was measured using the photometric method described by Ellman et al. [53]. A total reaction volume of 600 µL was prepared by adding 0.25 M sodium phosphate buffer solution pH 7.4, 1 mM 5,5′-dithiobis-2-nitrobenzoic acid solution (DTNB), enzymatic extract and 5 mM acetylthiocholine (chloride ATCh). After 10 min, the reaction was stopped with acetone, and the yellow color corresponding to thiocholine-DTNB complex was followed at room temperature at 412 nm. The enzyme activity was expressed as nmoles of ATCh hydrolyzed/min/mg protein.

#### 2.6.3. SOD Activity Determination

The method developed by Winterbourn et al. [54] was used to assess the SOD (E.C. 1.15.1.1) activity by monitoring its ability to inhibit the photoreduction of NBT. A total reaction volume of 1.4 mL containing 0.067 M potassium phosphate buffer pH 7.8, 0.1 M EDTA pH 7.8, enzymatic extract, 1.5 mM nitro blue tetrazolium (NBT) chloride and 0.12 mM riboflavin was prepared. The reaction mixture was exposed to light for ~30 min, and the blue formazan was followed at 560 nm. One SOD unit represented the amount of enzyme required to inhibit NBT reduction rate by 50%. SOD activity was expressed as units/mg protein.

#### 2.6.4. CAT Activity Determination

CAT (E.C. 1.11.1.6) activity was measured using the colorimetric protocol described previously by Sinha [55]. A volume of 125 µL enzymatic extract was allowed to react with an equal volume of 0.16 M H_2_O_2_ for 3 min at 37 °C. The reaction was stopped by adding 500 µL of potassium dichromate: acetic acid reagent. The tubes were incubated at 95 °C for 15 min, and the green color was read at 570 nm. One CAT unit is defined as 1 µmol of H_2_O_2_ consumed in 3 min. The enzyme activity was expressed as units/mg protein.

#### 2.6.5. GPX Activity Determination

GPX (E.C. 1.11.1.9) activity was measured using the protocol developed by Fukuzawa and Tokumura [56]. A total volume of 650 µL containing enzymatic extract, 0.25 M sodium phosphate buffer pH 7.4, 25 mM EDTA, and 0.4 M NaN_3_ was prepared and incubated at 37 °C for 10 min. An equal volume of 100 µM GSH and 50 mM H_2_O_2_ was added, and the tubes were incubated again at 37 °C for 10 min. The reaction was stopped with 730 µL of metaphosphoric acid 7%, and the tubes were centrifuged for 10 min at 960× *g*. Over 100 µL supernatant was added, as well as 0.3 M disodium phosphate solution and 0.04% DTNB. The GSH excess reacted with DTNB, and the yellow product was followed at 412 nm. One GPX unit was defined as the amount of enzyme required to oxidize 1 µmol of GSH/min. The enzyme activity was expressed as units/mg protein.

#### 2.6.6. Determination of the Total GSH Content

The GSH content was determined using the procedure described by Anderson [57] and modified by Salbitani et al. [58]. For this, 200 µL of supernatant was mixed with 1.1 mL of sodium phosphate buffer 0.25 M (pH 7.4) and 130 µL DTNB 0.04%. After 2 min of incubation at room temperature, the extinction was measured at 412 nm. The results were expressed as µg GSH/µg protein.

#### 2.6.7. Determination of Carbonylated Proteins Level

The method used to measure the level of carbonylated proteins is based on the reaction between carbonyl groups with 2,4-dinitrophenylhydrazine (DNPH) to form protein-bound 2,4- dinitrophenylhydrazones and was developed by Oliver et al. [59] and modified by Luo and Wehr [60]. A total of 1 mg protein was precipitated with 20% trichloroacetic acid (TCA, *w*/*v*, final concentration) and centrifuged at 960× *g* for 5 min. The pellet was resuspended in 0.2% DNPH solution (prepared in HCl 2N), and the tubes were incubated for 1 h at room temperature with periodic stirring. The samples were reprecipitated with 20% TCA and centrifuged at 960× *g* for 5 min. The pellet was washed three times with 1 mL ethanol-ethyl acetate (1:1, *v*/*v*) and left to dry at room temperature for 5 min. The precipitate was dissolved in guanidine hydrochloride 6M. The extinctions were measured at 370 nm, and the results were expressed as nmoles DNPH/mg protein.

#### 2.6.8. Determination of MDA Level

The MDA level was quantified using a High-Performance Liquid Chromatography (HPLC) method based on 2-thiobarbituric acid (TBA) assay, which was previously described by Domijan et al. [61] and modified through Vaides-Negustor and Mihasan [62]. A total of 50 µL of samples or standards was mixed with 12.5 µL NaOH 3M and incubated for 30 min at 60 °C in stirring conditions (300 rpm). A total of 0.5 mL of H_2_SO_4_ 98% and 0.25 mL of TCA 20% were added, and the tubes were centrifuged for 10 min at 960× *g*. A volume of 0.5 mL supernatant was mixed with 0.25 mL TBA 0.355%, and the tubes were incubated for 30 min at 90 °C in constant stirring and then centrifuged for 30 min at 20,800× *g*. HPLC analysis was performed by injecting a volume of 20 µL of sample or standard in Shimadzu Prominence system (Shimadzu Corporation, Kyoto, Japan) equipped with two LC-20AD pumps, SIL-20AC autosampler, CTO-20AC column oven, SPD M20A DAD detector and a Zorbax Eclipse XDB–C18 reverse-phase (RP) column (Agilent Technologies, Santa Clara, CA, USA) with a length of 250 mm and 3 µm particle size. The mobile phase was composed of methanol (Carl Roth, Karlsruhe, Germany): 30 mM monopotassium phosphate pH 6.7 (35:65) and was used at a flow rate of 1 mL/min for 20 min. The standard curve was made using different concentrations of 1,1,3,3-tetraethoxypropane (TEP, Sigma-Aldrich, Darmstadt, Germany). The TEP- or MDA-TBA pink adduct was detected at 532 nm and eluted at 9.5 ± 0.2 min. The peak area or height was taken into consideration in the final calculation, and the results were expressed as µmoles MDA/l.

### 2.7. RNA Isolation and Real Time Quantitative PCR (qRT-PCR)

The expression of *bdnf*, *arc* and *il-1β* genes in the hippocampus was assessed using RT-qPCR as previously described by Postu et al. [40] and Ionita et al. [63]. Total RNA was isolated from ~50 mg frozen tissue using SV Total RNA Isolation System kit (Promega, Madison, WI, USA) according to the manufacturer instructions. The reverse transcription and real-time amplification were carried out in a single-step amplification reaction using GoTaq^®^ 1-Step RT-qPCR System (Promega, Madison, USA) on a 5-plex HRM Rotor-Gene 6000 (Corbett, CA, USA) rotary real-time PCR machine. The reaction volume was 10 µL and contained the following: GoTaq^®^ Probe, qPCR Master Mix 2X (Promega, Wi, USA), GoScript™ RT Mix for 1-Step RT-qPCR 50X, 300 nM of pre-designed specific primers for *Rattus norvegicus* (Table 2), 100 ng of total RNA template and Nuclease-free water up to volume. Data were analyzed using Rotor-Gene Q-Pure Detection Software v. 2.2.3. (Qiagen, CA, USA).

### 2.8. Statistic Analysis

The behavioral, biochemical, and genetic data were statistically analyzed by two-way analysis of variance (ANOVA) and Tukey’s post hoc multiple comparison test using GraphPad Prism software v8.3 for Windows (La Jolla, CA, USA), considering the treatment as factor. The data are expressed as means ± standard error of mean (S.E.M.). A statistically significant difference was considered when *p* < 0.01. Pearson correlation coefficient (r) was used to correlate several behavioral and biochemical parameters with lipid peroxidation product (MDA).

## 3. Results and Discussion

### 3.1. Molecular Docking Simulations

The α7 subtype of nAChRs is highly expressed in the hippocampus and is involved in cognitive functions of the CNS, such as memory formation, and its modulation is emerging as a therapeutic approach for cognitive disorders, including AD [64]. In the AD brain, the ACh level is limited, and the loss of α7 nAChRs in the hippocampus was correlated with progressive loss of cognitive functions [64,65]. It has been demonstrated that many features of the pro-cognitive effects of NIC and its memory-enhancing potential are mediated through α7 nAChRs [64]. AChBP is a homopentameric water-soluble protein secreted by the glial cells of *Lymnaea stagnalis* with similar pharmacological properties to the homomeric α7 subtype of the nAChRs, including an affinity for ACh and high affinity for NIC [32,66]. AChBP shows a high sequence similarity (15–28% identity) to all ligand-gated ion channels, and for this reason, its crystal structure is used as a model for the extracellular domain of the pentameric nAChRs [32]. AChBP was co-crystallized with NIC bound in all five identical binding sites, located at the boundary between subunits [32,64]. The residues involved in the NIC binding site of AChBP were previously identified and described by Celie et al. [32]. To take into consideration a certain compound as a therapeutic agent for AD, it must have several NIC-like effects, especially in terms of nAChRs modulation [66]. In the present study, we conceived an in silico experiment in which we evaluated the binding potential of the two structural-related nicotinic derivatives, COT, and 6HLN, in the binding site of AChBP. To simplify the docking process, only two subunits that form the NIC binding site were kept. The best three binding poses of the ligands were selected based on the RMSD, binding energy, and ligand efficiency. We first docked (*S*)-nicotine in the binding cavity of AChBP, and we compared its orientation with that observed experimentally by Celie et al. [32] (Figure S1A (Supplementary Materials)). The very good fit between computationally obtained (*S*)-nicotine orientation and the one obtained experimentally (RMSD of 0.29 Å for 12 superimposed atoms of the ligand, Figure S1B (Supplementary Materials), Table 3) indicate that the docking method used is reliable. We further docked the (*S*)-cotinine and (*S*)-6-hydroxynicotine into the AChBP binding site, and their orientation and affinity, expressed as binding energy, were compared with the experimental data obtained for NIC. The theoretical binding pose of (*S*)-cotinine was different compared to NIC (RMSD of 0.27 Å for 9 superimposed atoms of which, only 6 were in a perfect match). Moreover, this compound showed a binding energy similar to that of (*S*)-nicotine (−6.6 for COT and −6.59 for NIC, Table 3) and ADT identified a hydrogen bond formed between the hydroxyl group of COT and Y89 residue located on the principal subunit of AChBP (Figure S1C). (*S*)-6-Hydroxynicotine showed a similar orientation in the binding cavity of AChBP with the one observed experimentally for NIC with an RMSD of 0.25 Å for 12 superimposed atoms of the ligand. In addition, our results indicated lower binding energy of (*S*)-6-hydroxynicotine (theoretical binding energy of −7.17) compared to (*S*)-nicotine (theoretical binding energy of −6.59), thus suggesting a higher affinity of 6HLN to AChBP than NIC (Table 3). ADT also identified a hydrogen bond formed between (*S*)-6-hydroxynicotine and M114 residue located on the complementary subunit of AChBP (Figure S1D). The ligand–receptor interactions analysis revealed that all ligands contact with Y89, W143, Y192 residues on the principal subunit (+) and with M114 residue on the complementary subunit (−) (Figure S2 (Supplementary Materials)). These results indicate that 6HLN and COT could bind to the α7 nAChRs with similar or higher affinity than NIC. The RMSD values are varying between the ligands because of the extra oxygen atom of COT and 6HLN that could not be overlapped with any of nicotine atoms. The RMSD values are varying between the conformations of the same ligand because there is a slight difference in the ligand orientation compared to the native molecule of nicotine. The conformations obtained by us have an orientation similar to that of the native nicotine molecule, but do not overlap perfectly.

**Figure 3:**
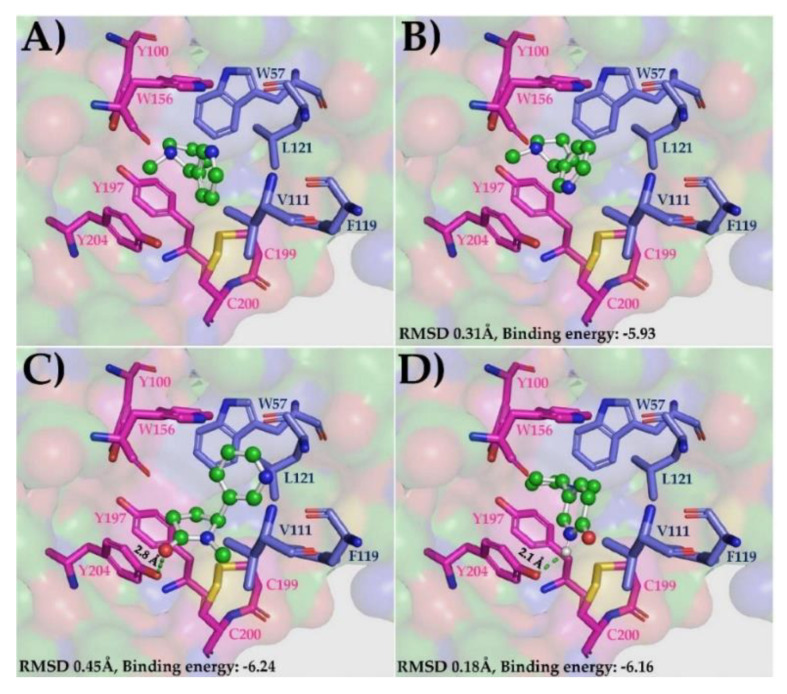
The native position of the NIC molecule in the α4-β2 site of α4β2 nAChRs (3α:2β, 6CNK) (**A**) and the best theoretical binding positions of (S)-nicotine (**B**), (S)-cotinine (**C**) and (S)-6-hydroxynicotine (**D**). The ligands are displayed as ball and sticks, the NIC-binding site-residues are shown as sticks (the residues with side chain colored in magenta belong to α4 subunit while the residues with side chain in dark blue belongs to β2 subunit), the hydrogen bonds are represented as green dashed lines and the rest of the receptor as molecular surface.

The α4β2 subtype of nAChRs is the most abundant isoform in the human brain and the main target in NIC addiction [33]. These receptors are involved in higher cognitive functions, such as attention, learning, and memory. According to post-mortem studies, their expression is in decline in more advanced stages of AD [67]. α4β2 nAChRs are assembled in two functional stoichiometries of α- and β-subunits: 2α:3β and 3α:2β [33]. The next step of this experiment was to evaluate and compare the binding potential of the nicotinic derivatives, COT and 6HLN, in two allosteric binding sites of the human α4β2 nAChRs (3α:2β): α4-α4 and α4-β2 respectively. The orientation of the NIC in the α4-α4 and α4-β2 sites, as well as the specific roles of the residues with which it interacts in the binding cavity (Figure S3A (Supplementary Materials)), were previously described by Walsh et al. [33]. Like AChBP, we kept only those subunits that formed the binding site of NIC to simplify the docking process. First, we docked (*S*)-nicotine in the α4-α4 binding site, and we compared its orientation with that observed experimentally for NIC by Walsh et al. [33]. The low RMSD value obtained for (*S*)-nicotine (0.23 Å for 12 superimposed atoms) confirm again that the docking method and parameters used are reliable (Figure S3B (Supplementary Materials)). We further docked the nicotinic derivatives in the α4-α4 site and we compared their orientation and affinity with that of NIC. RMSD calculation revealed that the theoretical binding pose of (*S*)-cotinine (Figure S3C (Supplementary Materials)) and (*S*)-6-hydroxynicotine (Figure S3D) was very similar to the NIC orientation obtained experimentally (RMSD of 0.4 Å for COT and 0.28 Å for 6HLN corresponding to 12 superimposed atoms). The interaction energies calculated by ADT showed that the nicotinic derivatives, especially (*S*)-6-hydroxynicotine, would be more tightly bound in α4-α4 site compared to (*S*)-nicotine (theoretical binding energy of −5.65 for COT, −5.71 for 6HLN and 5.5 for NIC, Table 3). This high affinity of 6HLN may be due to an extra hydrogen bond formed with a Y204 residue located on the principal α4 side of the subunit interface. In the α4-α4 site, the ligands interacted with Y100 and W156 residues on the principal side (α4+) and T126 residue on the complementary side (α4-) (Figure S4). In a similar way, the ligands were also docked in the α4-β2 interface. As in previous situations, we found a good overlap between the 12 atoms of (*S*)-nicotine and its orientation observed experimentally, resulting in an RMSD of 0.31 Å (Figure 3B). The theoretical binding position obtained for (S)-cotinine in α4-β2 site was quite different from NIC orientation determined experimentally (Figure 3C), resulting in an RMSD value of 0.45 Å for 10 superimposed atoms of which only seven were in a perfect match. However, in this putative pose, (*S*)-cotinine showed lower binding energy than (*S*)-nicotine (−6.24 for COT and 5.93 for NIC, Table 3), thus suggesting a higher affinity of this compound to the α4-β2 site than its parent molecule. This high affinity of COT could be explained by the additional hydrogen bond identified by ADT between the hydroxyl group of COT and the Y204 residue located on the α4 subunit. We have also found that the theoretical binding pose of (*S*)-6-hydroxynicotine was very similar with an NIC orientation determined experimentally (RMSD of 18 Å for 12 superimposed atoms, Figure 3D). In this putative pose, (*S*)-6-hydroxynicotine bonded in the α4-β2 site with a higher affinity than NIC (theoretical binding energy of -6.16 for 6HLN and −5.93 for NIC, Table 3) and, similar to COT, it formed an extra hydrogen bond with Y204 residue on the α4 subunit. According to Ligplot+ software, the ligands interacted with W156 and Y204 residues on α4 subunit and with L121 residue on β2 subunit (Figure S5 (Supplementary Materials)). In order to highlight a possible affinity (preference) of COT and 6HLN for a certain type of site, we compared the theoretical binding energies of the ligands docked in α4-α4 site with those of the ligands docked in α4-β2 site. Our results indicated that for each ligand, the theoretical binding energies corresponding to α4-β2 site are lower than those calculated for the α4-α4 site (Table 3), thus suggesting a potential preference of the nicotinic derivatives over nAChRs, which contains a greater number of α4-β2 type interfaces. These results suggest that 6HLN and COT could bind in both allosteric binding sites of α4β2 nAChRs with higher affinity than NIC and display a preference towards the α4-β2 interface.

Our results agree with those from the literature and indicated that both COT and 6HLN could bind to the α7 and α4β2 subtypes of nAChRs. Moreover, the docking simulations performed on α4β2 nAChRs revealed a higher binding affinity of these compounds in the α4-β2 interface to the detriment of the α4-α4 interface. Using a set of in silico tools, Mihasan et al. [66] screened all the metabolic intermediates resulting from the NIC degradation pathway located on the pAO1 megaplasmid of *P. nicotinovorans*, for their ability to bind AChBP. Their findings indicated that 6HLN could bound more tightly to AChBP compared to NIC due to an extra hydrogen bond formed between the hydroxyl group of 6HLN and Y185 residue of AChBP [66]. COT is a weak agonist of α7 nAChRs, having 100 times less affinity for α7 nAChRs than NIC. However, accumulated evidence suggested that COT might act as a type I positive allosteric modulator (PAM) of α7 nAChRs, enhancing their activation by facilitating the effects of endogenous agonists, such as ACh or the efficacy of AChE inhibitors, such as DNP [23,24]. Terry et al. [68] suggested that COT might sensitize α7 nAChRs to low levels of ACh and could be used as an adjunctive agent to improve the dose range of DNP. Several lines of evidence showed that COT stimulates the activation of protein kinase B (Akt)/glycogen synthase kinase 3β (GSK3β) and Akt/postsynaptic density protein 95 (PSD95)-cAMP responsive element-binding protein (CREB) pathways in the hippocampus and cortex of AD mice [29,30]. These pathways support neuronal survival and synaptic plasticity, processes that underlie memory and learning, and are located downstream of α7 nAChRs. Thus, their enhanced activation has been attributed to the ability of COT to positively modulate these receptors [23]. The activation of Akt pathway could trigger a cascade of signaling pathways [23], leading to the stimulation of pro-survival proteins, such as Bcl-2 or CREB [69] and the inhibition of pro-apoptotic protein c-Jun N-terminal kinase (JNK) [70]. Moreover, the α7 nAChRs could support the synaptic plasticity and cognition by activating the protein kinases phosphoinositide-3 kinase (PI3K), Akt, extracellular signal-regulated kinase 1/2 (ERK1/2) and the transcription factor CREB [23]. GSK3β is a proline-directed kinase and is considered to be the main kinase that phosphorylates tau protein, the major component of NFTs observed in AD [71]. It has been demonstrated that Akt pathway stimulation by COT inhibits GSK3β by phosphorylation in the brains of AD mice [30,72] and restrained mice [73].

Electrophysiological studies showed weak or no agonistic effect of COT on α4β2 nAChRs [68,74]. However, a previous study performed on monkeys showed that COT interacted with α4β2 subtype of nAChRs and stimulated the dopamine release from caudate synaptosomes [75]. Moreover, COT was found to modulate sensory inhibition through α4β2 nAChRs in a mouse model of schizophrenia [76]. Fox et al. [77] demonstrated that COT exposure increased the number of α4β2 nAChRs on the plasma membrane and caused a redistribution of intercellular receptors. Their findings also indicated that COT altered the α4β2 nAChRs assembly to favor the 2α:3β stoichiometry. It has been demonstrated that in α4β2 nAChRs (3α:2β), the high-sensitivity agonist sites are found at the α4-β2 subunit interfaces while the α4-α4 interface might serve as a low-sensitivity agonist site [33,78]. However, to obtain a full response, the low-sensitivity site is needed to be occupied [78].

Consistent with these studies, our data indicated that COT and 6HLN could bind and modulate the α7 and α4β2 nAChRs, leading to an improvement in cholinergic neuronal transmission and cognitive abilities in different dementia-related conditions, such as AD.

### 3.2. Effects of Nicotinic Derivatives on Cognitive Functions

Based on the results obtained in silico, we further used in vivo tasks to investigate the impact of nicotinic derivatives on cognitive abilities in an animal model of AD induced by i.c.v. injection of Aβ_25-35_ peptide. The Aβ_25-35_ peptide is a fragment of 11 amino acids residues from the full-length Aβ_1-42_ peptide and was observed in the extracellular aggregates of Aβ in the brain of AD patients [79]. This fragment retains most of the physical and biological properties of its parent molecule, including toxicity to neurons [80,81]. The brain injection of Aβ_25-35_ peptide was reported to induce behavioral changes, especially in terms of memory, in rodents, and can be used to induce an animal model of AD [41,82,83].

**Figure 4:**
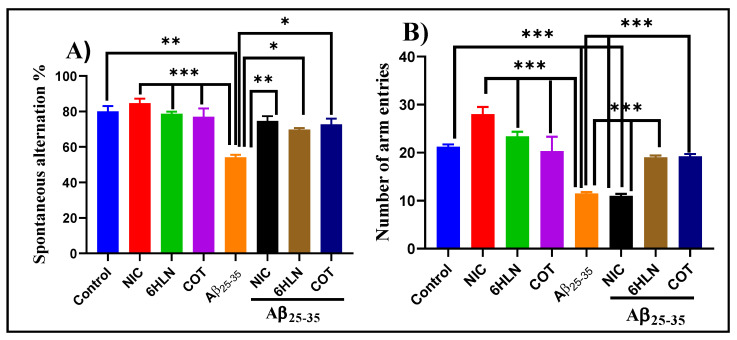
Effects of NIC, 6HLN and COT (0.3 mg/kg, b.w., i.p.) administration in Aβ_25-35_-treated rats on (**A**) spontaneous alternation percentage and (**B**) the number of arm entries in the Y-maze task. Values are expressed as means ± S.E.M. (*n* = 6 animals per group). ANOVA analysis identified overall significant differences between groups in (**A**) F(4,10) = 16.5, *p* < 0.0002 and (**B**) F(4,15) = 130.8, *p* < 0.0001. For Tukey’s *post hoc* analyses–(**A**) ** Control vs. Aβ_25-35_, *** NIC vs. Aβ_25-35,_ 6HLN vs. Aβ_25-35_, COT vs. Aβ_25-35_*: p* < 0.0001, ** Aβ_25-35_ vs. NIC + Aβ_25-35_: *p* < 0.001, * Aβ_25-35_ vs. 6HLN + Aβ_25-35_, Aβ_25-35_ vs. COT + Aβ_25-35_: *p* < 0.01; (**B**)*** Control vs. Aβ_25-35_, Control vs. NIC + Aβ_25-35_, NIC vs. Aβ_25-35_, 6HLN vs. Aβ_25-35_, COT vs. Aβ_25-35_, Aβ_25-35_ vs. 6HLN + Aβ_25-35_, NIC + Aβ_25-35_ vs. 6HLN + Aβ_25-35_, Aβ_25-35_ vs. COT + Aβ_25-35_, NIC + Aβ_25-35_ vs. COT + Aβ_25-35_: *p* < 0.0001.

Y-maze is a hippocampus-dependent task and was used to assess the effects of COT and 6HLN (0.3 mg/kg, b.w., i.p.) on short-term spatial recognition memory in an Aβ_25-35_-induced rat model of AD. As is depicted in Figure 4A, the infusion of Aβ_25-35_ peptide in the rat’s hippocampus impaired the spatial recognition memory by significantly reduced (*p* < 0.001) the spontaneous alternation percentage compared to the sham-operated control group. Administration of 6HLN and COT in Aβ_25-35_-treated rats significantly improved (*p* < 0.01 for 6HLN and COT, respectively) the cognitive performances of the animals compared to the group treated with the peptide alone. Moreover, Aβ_25-35_ also caused a severe reduction in locomotor activity by significantly decreasing (*p* < 0.0001 vs. control group) the total number of arm entries (Figure 4B). This hypolocomotor effect of Aβ_25-35_ peptide suggests that the movement reduction in rats could have an impact on the quantification of the spontaneous alternation percentage. In addition, the post hoc comparison indicated that coadministration of 6HLN or COT antagonized the effect of Aβ_25-35_ peptide, by significantly increasing the total number of arm entries in Y-maze task (*p* < 0.0001). These results indicate that chronic administration of 6HLN and COT to Aβ_25-35_-treated rats improved the short-term spatial recognition memory within the Y-maze task.

**Figure 5:**
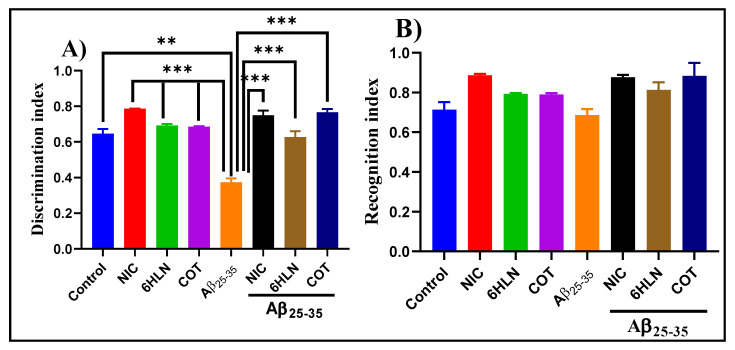
Effects of NIC, 6HLN and COT (0.3 mg/kg, b.w., i.p.) administration in Aβ_25-35_-treated rats on (**A**) discrimination and (**B**) recognition indices within NOR task. Values are expressed as means ± S.E.M. (*n* = 6 animals per group). ANOVA analysis revealed overall significant differences between groups in (**A**) F(4,10) = 37.45, *p* < 0.0001. For Tukey *post hoc* analyses–(**A**) ** Control vs. Aβ_25-35_: *p* < 0.01; *** NIC vs. Aβ_25-35_, 6HLN vs. Aβ_25-35_, COT vs. Aβ_25-35_, Aβ_25-35_ vs. NIC + Aβ_25-35_, Aβ_25-35_ vs. 6HLN + Aβ_25-35_, Aβ_25-35_ + COT + Aβ_25-35_: *p* < 0.0001.

Within NOR task, we evaluated the effects of NIC (0.3 mg/kg, b.w., i.p.) and nicotinic derivatives, COT, and 6HLN (0.3 mg/kg, b.w., i.p.), on recognition memory consolidation in the rat model of AD induced by Aβ_25-35_. As shown in Figure 5A, compared to the control group, Aβ_25-35_ peptide-treated rats displayed a lower value of DI, thus suggesting a shorter period of exploratory time of the novel object. However, the chronic treatment with 0.3 mg/kg of NIC, 6HLN, and COT was found to be effective in Aβ_25-35_-treated rats, increasing the DI to a value closer to the control group. Regarding RI, the main index of retention, ANOVA analysis revealed no overall significant differences between the experimental groups (Figure 5B). These results indicate that 6HLN and COT produced an improving effect of recognition memory in a rat model of AD induced by Aβ_25-35_ peptide.

**Figure 6:**
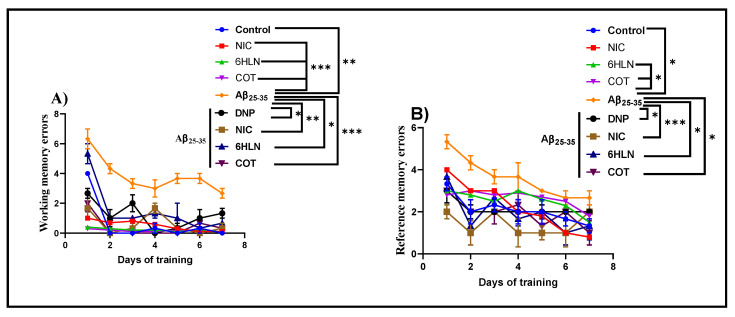
Effects of NIC, 6HLN and COT (0.3 mg/kg, b.w., i.p.) administration in Aβ_25-35_-treated rats on (**A**) working memory errors and (**B**) reference memory errors in RAM task. Values are expressed as means ± S.E.M. (*n* = 6 animals per group). ANOVA analysis identified overall significant differences between experimental groups in (**A**) F(5.36) = 8.082, *p* < 0.0001 and (**B**) F(5.36) = 7.542, *p* < 0.0001. For Tukey *post-hoc* analyses—(**A**) ** Control vs. Aβ_25-35_: *p* < 0.01; *** NIC vs. Aβ_25-35_, 6HLN vs. Aβ_25-35,_ COT vs. Aβ_25-35_: *p* < 0.0001; * Aβ_25-35_ vs. DNP + Aβ_25-35_, Aβ_25-35_ vs. 6HLN + Aβ_25-35_: *p* < 0.01; ** Aβ_25-35_ vs. NIC + Aβ_25-35_: *p* < 0.001; *** Aβ_25-35_ vs. COT + Aβ_25-35_: *p* < 0.0001; (**B**) * Control vs. Aβ_25-35_, 6HLN vs. Aβ_25-35_, COT vs. Aβ_25-35_: *p* < 0.01; * Aβ_25-35_ vs. DNP + Aβ_25-35_, Aβ_25-35_ vs. 6HLN + Aβ_25-35_; Aβ_25-35_ vs. COT + Aβ_25-35_: *p* < 0.01; *** Aβ_25-35_ vs. NIC + Aβ_25-35_: *p* < 0.0001.

RAM task was further used to investigate whether chronic treatment with 0.3 mg/kg of COT and 6HLN could have any impact on spatial memory, expressed as working and reference memory, in Aβ_25-35_ peptide-induced rat model of AD. Figure 6A shows that i.c.v. infusion of Aβ_25-35_ peptide caused a significant increase (*p* < 0.001) in the number of working memory errors compared to the control group, thus suggesting an impairment of short-term memory. Similarly, but to a lesser extent, Aβ_25-35_ peptide also caused an impairment of long-term memory by significantly increasing (*p* < 0.01) the number of reference memory errors (Figure 6B). DNP is a commonly prescribed therapeutic agent of AD and was used as a positive control. Acute administration of DNP (5mg/kg, b.w., i.p.) significantly improved (*p* < 0.01) both short- and long-term memory in rats treated with Aβ_25-35_ peptide. Nevertheless, post hoc comparison indicated that chronic administration of nicotinic derivatives in Aβ_25-35_-treated rats significantly reduced the working memory errors (*p* < 0.01 for 6HLN and *p* < 0.0001 for COT, Figure 6A) and reference memory errors (*p* < 0.01 for 6HLN and COT, Figure 6B). These results suggest that 6HLN and COT improved spatial memory in Aβ_25-35_ peptide-induced AD-like rats.

The behavioral results obtained in this study are in perfect agreement with those from the literature and indicated that 6HLN and COT ameliorated the cognitive deficits in a rat model of AD induced by i.c.v. infusion of Aβ_25-35_ peptide.

Hritcu et al. [84] demonstrated for the first time that chronic administration of 6HLN (0.3 mg/kg, b.w., i.p.) to normal Wistar rats sustained spatial memory, especially short-term memory and working memory, without affecting long-term memory. Moreover, the effects of 6HLN treatment on memory impairments in a rat model of AD induced by scopolamine (SCOP, 0.7 mg/kg, b.w., i.p.), an antagonist of muscarinic acetylcholine receptors, were also evaluated [85]. The results indicated that the administration of 6HLN (0.3 mg/kg, b.w., i.p.) ameliorated the SCOP-induced memory deficits by improving spatial working memory within the Y-maze task and memory formation in RAM task [85]. Recently, in a side comparation study with NIC, the cognitive effects of 6HLN were investigated in a rat model of AD induced by chlorisondamine (CHL, 10 mg/kg, b.w., i.p.), a nAChRs antagonist [28]. It has been shown that 6HLN injection to CHL-treated rats selectively enhanced spatial memory formation in Y-maze and RAM tasks to a greater extent than NIC. Taken together, these studies suggested that 6HLN could play a key role in spatial memory restoration, mainly in rodents with cholinergic deficits.

Numerous studies indicated that COT acts as a cognitive enhancer in animal models of several mental health conditions, such as AD [29,30], Parkinson’s disease (PD) [86], schizophrenia [87] or post-traumatic stress disorder (PTSD) [88]. Moreover, it has been shown that COT improved both visual and working memory in an animal model of chemotherapy-induced cognitive impairment [89] and also improved cognition and memory in the mouse model of Fragile X syndrome [90]. A recent study indicated that both *R*-(+) and *S*-(−) isomers of COT improved the recognition memory of the rats by enhancing the effective dose range of cholinergic compounds, such as DNP, a therapeutic agent of AD [68]. In an extensive study performed by Echeverria et al. [30], COT impact on cognitive impairments was investigated in transgenic mice (Tg 6799) that develop AD very fast by expressing five familial AD mutations. Their findings indicated that chronic treatment with COT (2.5 mg/kg, via gavage) prior and, subsequently, AD development, improved both working and reference memories. Furthermore, the COT effects were also investigated when the treatment was administered in the same strain of AD mice, but in more advanced stages of pathology’s development and a double dose. Patel et al. [29] demonstrated that COT ameliorated the effects of AD-like pathology by improving spatial and working memory performances in the radial arm water maze test. The cognitive impairment in AD was correlated with an increase in aggregated Aβ peptide in the brain [91]. Salomon et al. [92] demonstrated in vitro that COT inhibited amyloidosis, to a lesser extent than NIC, by binding Aβ peptide in an α-helical structure, thus preventing conversion in β-sheet. This anti-aggregation effect of COT might occur due to a high bond affinity [93] between COT moieties and histidine residues of Aβ [92]. In this direction, Echeverria et al. [30] showed through molecular dynamics simulations that important changes in Aβ_1-42_ structure might occur due to a possible interaction between COT and H6, Y10 and H14 residues that prevents aggregation. Burgess et al. [94] demonstrated that COT inhibited Aβ neurotoxicity on embryonic cortical neurons only when it was included in pre-aggregation solutions of Aβ_1-42_ but not when it was added later in the media, thus suggesting that the COT is neuroprotective against Aβ-induced cortical cell death by preventing its aggregation. This neuroprotective activity of COT was not blocked by mecamylamine, a nAChRs antagonist, suggesting that this beneficial effect of COT is nAChRs-independent [94]. In addition, COT diminished Aβ plaque deposition in the cortex and hippocampus of AD mice by reducing the number and size of Aβ plaques and decreasing the insoluble Aβ levels [29,30]. Because the Aβ_25-35_ peptide used in the current study was already in an aggregated form (fibrils) and lacked the residues reported to be involved in the interaction with COT, it is unlikely that the pro-cognitive effects of COT in Aβ_25-35_-induced rat model of AD are due to the anti-aggregation potential of this compound. However, we believe that the promnesic effects of COT might be nAChRs-related, as their modulation could stimulate the activation of several pathways located downstream of nAChRs which underlies memory and learning processes. Due to the close structural similarity with COT, we considered that the cognitive-enhancing ability of 6HLN in Aβ_25-35_- treated rats is also related to nAChRs modulation. Consistent with these studies, we demonstrated that 6HLN and COT mitigated the cognitive deficits caused by i.c.v. administration of Aβ_25-35_ peptide in rats.

### 3.3. Effects of Nicotinic Derivatives on AChE Specific Activity

**Figure 7:**
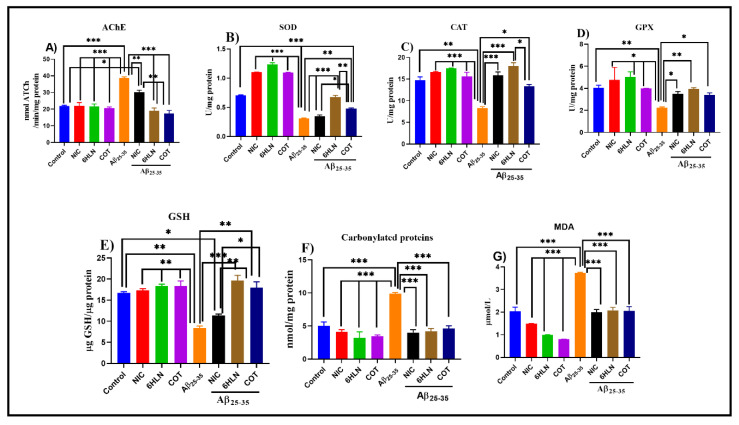
Effects of NIC, 6HLN and COT (0.3 mg/kg, b.w., i.p.) administration in Aβ_25-35_-treated rats on (**A**) AChE-specific activity and oxidative status, represented by (**B**) SOD, (**C**) CAT and (**D**) GPX-specific activities along with the content of (**E**) GSH, (**F**) carbonylated proteins and (**F**) MDA in the hippocampus. Values are expressed as means ± S.E.M. (*n* = 6 animals per group). ANOVA analysis revealed overall significant differences between groups in (**A**) F(4,10) = 48.92, *p* < 0.0001, (**B**) F(4,10) = 100.7, *p* < 0.0001, (**C**) F(4.10) = 30.49, *p* < 0.0001, (**D**) F(4,10) = 18.15, *p* < 0.0001, (**E**) F(4,10) = 30.06, *p* < 0.0001, (**F**) F(4,10) = 32.55, *p* < 0.0001 and (**G**) F(4,10) = 26.87, *p* < 0.0001. For Tukey *post hoc* analyses – (**A**)*** Control vs. Aβ_25-35_, NIC vs. Aβ_25-35_, 6HLN vs. Aβ_25-35_, COT vs. Aβ_25-35_, Aβ_25-35_ vs. 6HLN + Aβ_25-35_, Aβ_25-35_ vs. COT + Aβ_25-35_: *p* < 0.0001, ** Aβ_25-35_ vs. NIC + Aβ_25-35_, NIC + Aβ_25-35_ vs. 6HLN + Aβ_25-35_, 6HLN + Aβ_25-35_ vs. COT + Aβ_25-35_: *p* < 0.001, * Control vs. NIC + Aβ_25-35_ vs: *p* < 0.01; (**B**) *** Control vs. Aβ_25-35_, Control vs. COT + Aβ_25-35_, NIC vs. Aβ_25-35_, 6HLN vs. Aβ_25-35_, COT vs. Aβ_25-35_, Aβ_25-35_ vs. NIC + Aβ_25-35_, Aβ_25-35_ vs. 6HLN + Aβ_25-35_: *p* < 0.0001, ** Aβ_25-35_ vs. COT + Aβ_25-35_, 6HLN + Aβ_25-35_ vs. COT + Aβ_25-35_: *p* < 0.001, *NIC + Aβ_25-35_ vs. COT + Aβ_25-35_: *p* < 0.01; (**C**) *Control vs. Aβ_25-35_: *p* < 0.001, *** NIC vs. Aβ_25-35_, 6HLN vs. Aβ_25-35_, COT vs. Aβ_25-35_, Aβ_25-35_ vs. NIC + Aβ_25-35_, Aβ_25-35_ vs. 6HLN + Aβ_25-35_: *p* < 0.0001, * Aβ_25-35_ vs. COT + Aβ_25-35_, 6HLN + Aβ_25-35_ vs. COT + Aβ_25-35_: *p* < 0.01; (**D**) ** Control vs. Aβ_25-35_, Aβ_25-35_ vs. 6HLN + Aβ_25-35_: *p* < 0.001, *NIC vs. Aβ_25-35_, 6HLN vs. Aβ_25-35_, COT vs. Aβ_25-35_, Aβ_25-35_ vs. NIC + Aβ_25-35_, Aβ_25-35_ vs. COT + Aβ_25-35_: *p* < 0.01; (**E**) *Control vs. NIC + Aβ_25-35_, NIC + Aβ_25-35_ vs. COT + Aβ_25-35_: *p* < 0.01, ** Control vs. Aβ_25-35_, NIC vs. Aβ_25-35_, 6HLN vs. Aβ_25-35_, COT vs. Aβ_25-35_, Aβ_25-35_ vs. COT + Aβ_25-35_, NIC + Aβ_25-35_ vs. 6HLN + Aβ_25-35_: *p* < 0.001, *** Aβ_25-35_ vs. 6HLN + Aβ_25-35_: *p* < 0.0001; (**F**) *** Control vs. Aβ_25-35_, NIC vs. Aβ_25-35_, 6HLN vs. Aβ_25-35_, COT vs. Aβ_25-35_, Aβ_25-35_ vs. NIC + Aβ_25-35_, Aβ_25-35_ vs. 6HLN + Aβ_25-35_, Aβ_25-35_ vs. COT + Aβ_25-35_: *p* < 0.0001; (**G**) *** Control vs. Aβ_25-35_, NIC vs. Aβ_25-35_, 6HLN vs. Aβ_25-35_, COT vs. Aβ_25-35_, Aβ_25-35_ vs. NIC + Aβ_25-35_, Aβ_25-35_ vs. 6HLN + Aβ_25-35_, Aβ_25-35_ vs. COT + Aβ_25-35_: *p* < 0.0001.

The cholinergic hypothesis claimed that the reduction in ACh synthesis is the main cause of AD. AChE catalyzes the hydrolysis of ACh in choline and acetate ions. Therefore, increasing the level of ACh in the brain by inhibiting the biological activity of AChE represents a therapeutic approach in AD [95]. Here, we evaluated the effects of 6HLN and COT on brain AChE specific activity in Aβ_25-35_-treated rats. Our results indicated that brain delivery of Aβ_25-35_ peptide significantly increased (*p* < 0.0001) the AChE specific activity in the rat’s hippocampus compared to the control group (Figure 7A). However, 6HLN and COT (0.3 mg/kg, b.w., i.p.) were found to be more effective than NIC (*p* < 0.001), significantly reducing (*p* < 0.0001) the AChE-specific activity in the hippocampus of the Aβ_25-35_-treated rats to a level close to control group (Figure 7A). These results suggest an anti-AChE profile of 6HLN and COT in an Aβ_25-35_ peptide-induced animal model of dementia.

### 3.4. Effects of Nicotinic Derivatives on Oxidative Status

Oxidative stress is a serious imbalance between reactive oxygen species (ROS) production and antioxidant defenses, and it was previously shown to contribute significantly to AD pathogenesis and progression [96]. During oxidative stress, ROS reacts with cellular biomolecules (lipids, sugars, proteins, and polynucleotides) inflicting oxidative damage [96,97]. Thereby, several defense systems are involved in preventing the increase in ROS production. These systems include non-enzymatic molecules, such as glutathione, as well as enzymatic scavengers of ROS, such as SOD, CAT, or GPX [97]. The oxidative damage can be detected by measuring specific products that result from this damage, such as MDA, the most investigated end product of lipid peroxidation, or carbonylated proteins, the most widely used marker of oxidative protein damage [97]. In AD, elevated oxidative stress was identified in brain regions rich in Aβ [98]. The ROS production can be catalyzed by redox-active metal ions when they are bounded to Aβ [99]. Aβ_25-35_ caused oxidative stress in different animal cell cultures [100,101] and rat hippocampus [82]. We further evaluated the effects of nicotinic derivatives on oxidative stress in the Aβ_25-35_ peptide-induced rat model of AD by measuring the SOD, CAT and GPX specific activities and the levels of GSH, MDA and carbonylated proteins in rat hippocampus.

Our results indicated that i.c.v. administration of Aβ_25-35_ peptide significantly decreased the SOD (*p* < 0.0001, Figure 7B), CAT (*p* < 0.001, Figure 7C) and GPX (*p* < 0.001, Figure 7D)-specific activities compared to sham-operated control group. Moreover, this effect of the peptide was accompanied by a significant decrease in GSH content (*p* < 0.001, Figure 7E) as well as an increase in the levels of MDA (*p* < 0.0001, Figure 7F) and carbonylated proteins (*p* < 0.0001, Figure 7G). However, chronic treatment (0.3 mg/kg, b.w., i.p.) with nicotinic derivatives, especially 6HLN, significantly restored the SOD (*p* < 0.0001 for 6HLN and *p* < 0.001 for COT, Figure 7B), CAT (*p* < 0.0001 for 6HLN and *p* < 0.01 for COT, Figure 7C) and GPX (*p* < 0.001 for 6HLN and *p* < 0.01 for COT, Figure 7D) specific activities in the hippocampus of Aβ_25-35_-treated rats. In addition, post hoc analyses revealed that the treatment significantly increased the GSH content (*p* < 0.001 for 6HLN and *p* < 0.01 for COT, Figure 7E) and significantly reduced the MDA (*p* < 0.0001, Figure 7F) and carbonylated protein levels (*p* < 0.0001, Figure 7G) compared to the group treated with peptide alone.

Our results are in line with previous findings and indicated that 6HLN and COT displayed neuroprotective effects against Aβ_25-35_ peptide-induced oxidative stress. Through the quantitative-structure-analysis relationship (QSAR) equation [66] and ferric-reducing ability of plasma (FRAP) [102] assay, it has been suggested that the antioxidant properties of 6HLN are superior to those of its parent molecule. These improved properties were attributed to the hydroxyl group that 6HLN has in addition to NIC [66,102]. Moreover, Hritcu et al. [84] demonstrated in vivo that chronic administration of 6HLN has positive effects on antioxidant status in normal Wistar rats by increasing the SOD and GPX specific activities and decreasing the MDA level in the temporal cortex. Furthermore, the antioxidant effects of 6HLN were also evaluated in two other rat models of AD. It has been shown that 6HLN reduced the oxidative stress induced by SCOP or CHL in the rat hippocampus restoring the SOD, CAT, and GPX specific activities, increasing the total content of GSH and decreasing MDA production [28,85].

An early study has shown that COT was able to suppress the production of the oxygen free radicals by neutrophils in smokers and non-smokers [103]. Soto-Otero et al. [104] demonstrated in vitro that COT increased the production of hydroxyl radicals (^•^OH) during autoxidation of 6-hydroxydopamine (6-OHDA), a neurotoxin widely used in experimental studies on PD pathogenesis. Additionally, COT had a similar effect on ^•^OH formation by Fenton reaction, only when COT was previously incubated with Fe^2+^, thus preventing the reaction. Furthermore, COT was able to reduce the MDA formation provoked by 6-OHDA autoxidation in rat brain mitochondria preparations [104]. However, conflicting results were obtained by Aguiar et al. [105], which showed that COT increased lipid peroxidation but also increased antioxidant capacity in rat hippocampus. Nevertheless, the authors suggested that this increase in oxidative stress by COT depends upon the dose and did not cause the corresponding cognitive impairments. In line with previous results, we found that 6HLN and COT exhibit an antioxidant profile, decreasing the oxidative stress in the brain of a rat model of AD.

**Figure 8:**
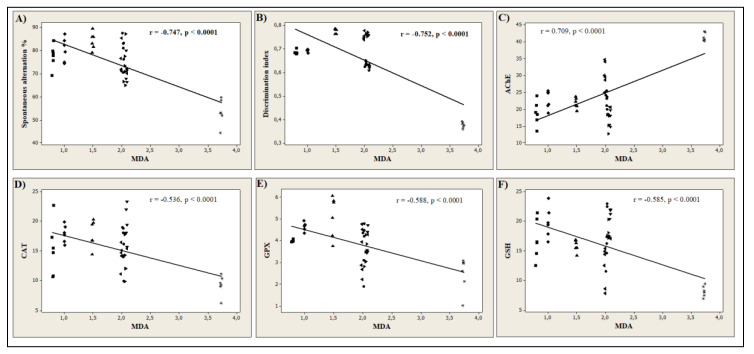
Pearson’s correlation between behavioral or biochemical parameters and the product of lipid peroxidation, MDA (*n* = 6): (**A**) Spontaneous alternation% vs. MDA (r = −0.747, *p* < 0.0001), (**B**) Discrimination index vs. MDA (r = −0.752, *p* < 0.0001), (**C**) AChE vs. MDA (r = 0.709, *p* < 0.0001); (**D**) CAT vs. MDA (r = −0.536, *p* < 0.0001); (**E**) GPX vs. MDA (r = −0.588, *p* < 0.0001); (**F**) GSH vs. MDA (r = −0.585, *p* < 0.0001) in Control (●), NIC (▲), 6HLN (♦), COT (■), Aβ_25-35_ (*), NIC + Aβ_25-35_ (◄), 6HLN + Aβ_25-35_ (▼) and COT + Aβ_25-35_ (►) groups. Data represent AChE (nmol ATCh/min/mg protein), CAT (U/mg protein), GPX (U/mg protein) and GSH (µg GSH/mg protein).

In the current study, the Pearson correlation coefficient (r) was used to quantify the linear association between different behavioral scores and biochemical parameters. Our results revealed that the spontaneous alternation percentage (*n* = 6, r = −0.747, *p* < 0.0001, Figure 8A) measured in Y-maze task, and the discrimination index (*n* = 6, r = −0.752, *p* < 0.0001, Figure 8B) determined in NOR task, strongly correlates with MDA, the product of lipid peroxidation. This suggests that the memory improvement of the rats by 6HLN or COT is well correlated with a decrease in the MDA level in the hippocampus. Moreover, we also correlated AChE and several defense systems, including CAT, GPX, and GSH, with MDA. Figure 8C shows a high positive correlation (*n* = 6, r = 0.709, *p* < 0.0001) between AChE and MDA, suggesting that a reduction in the AChE-specific activity is well correlated with a low level of lipid peroxidation. Regarding the antioxidant system, strong negative correlations were observed between CAT (*n* = 6, r = −0.536, *p* < 0.0001, Figure 8D), GPX (*n* = 6, r = −0.588, *p* < 0.0001, Figure 8E), GSH (*n* = 6, r = −0.585, *p* < 0.0001, Figure 8F) and MDA indicating that an increase in the activities of antioxidant enzymes and GSH content is well correlated with a reduction in MDA formation. The correlation coefficients obtained in the current study indicate that the antioxidant potential of 6HLN and COT is well correlated with their memory-enhancing ability and anti-AChE profile. In normal rats treated with 6HLN, Hritcu et al. [84] demonstrated a significant positive correlation between several behavioral parameters monitored in Y-maze or RAM and different endpoints of oxidative stress, thus suggesting that the behavioral performances of the rats might be related to the antioxidant properties of 6HLN. Similarly, the involvement of 6HLN in neuroprotection against SCOP was found to be correlated with an increase in cognitive performances and antioxidant defence along with a reduction in lipid peroxidation [85]. Echeverria et al. [30] suggested that the pro-cognitive profile of COT in AD mice might be explained by the ability of this compound to decrease Aβ levels and suppress its aggregation in the hippocampus and cortex. Moreover, Grizzell et al. [73] associated the antidepressant and nootropic effects of COT with an enhancement of synaptic density in the hippocampus, prefrontal, and entorhinal cortices of restrained mice. Pardo et al. [90] suggested that the cognitive-enhancing effects of COT are associated with inhibitory phosphorylation of GSK3β in the hippocampus of a mouse model of Fragile X syndrome. Finally, Zeitlin et al. [106] postulated that the enhancement of conditional fear extinction by COT might be mediated through ERK1/2 activation in the hippocampus of a mice model of PTSD. Coherent with these studies, we found that 6HLN and COT sustained memory formation and exhibited an antioxidant profile along with a decrease in brain AChE activity.

### 3.5. Effects of Nicotinic Derivatives on Gene Expression

#### 3.5.1. Effects of Nicotinic Derivatives on Bdnf Expression

Brain-derived neurotrophic factor (BDNF) is the most widely distributed neurotrophin in the CNS and is important for cellular growth, development, survival and synaptic activity [107,108]. All these actions are mediated by the selective binding of BDNF to the tyrosine kinase receptor B (TrkB) [108]. A reduced BDNF signaling through TrkB leads to spatial memory deficits [107,109]. Reduced BDNF mRNA and protein levels were identified in post-mortem brain samples of AD patients [107]. It has been suggested that Aβ could reduce BDNF levels by directly inhibiting its proteolytic conversion from pro-BDNF [110] and indirectly by interfering with its axonal transport [111]. As a compensatory response to the Aβ insult, BDNF seems to possess protective effects on neuronal toxicity induced by Aβ peptides in vitro and in vivo [112,113,114]. To evaluate the impact of nicotinic derivatives on neuroprotection, we investigated the *bdnf* gene expression in the Aβ_25-35_-treated rat’s hippocampus.

**Figure 9:**
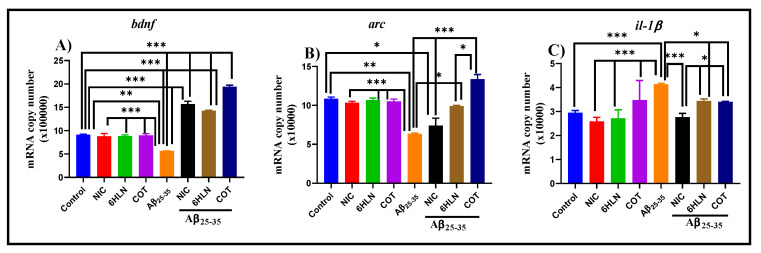
Effects of NIC, 6HLN and COT (0.3 mg/kg, b.w., i.p.) administration in Aβ_25-35_-treated rats on the mRNA copy number of (**A**) *bdnf*, (**B**) *arc* and (**C**) *il-1β* from the hippocampus. The values are expressed as means ± S.E.M. (*n* = 6 animals per group). ANOVA analysis identified general significant differences between groups in (**A**) F(4.10) = 291.7, *p* < 0.0001, (**B**) F(4.10) = 28.73, *p* < 0.0001 and (**C**) F(4,10) = 33.48, *p* < 0.0001. For Tukey post hoc analyses–(**A**) *** Control vs. NIC + Aβ_25-35_, Control vs. 6HLN + Aβ_25-35_, Control vs. COT + Aβ_25-35_, NIC vs. Aβ_25-35_, 6HLN vs. Aβ_25-35_, COT vs. Aβ_25-35,_ Aβ_25-35_ vs. NIC + Aβ_25-35_, Aβ_25-35_ vs. 6HLN + Aβ_25-35_, Aβ_25-35_ vs. COT + Aβ_25-35_, NIC + Aβ_25-35_ vs. COT + Aβ_25-35_, 6HLN + Aβ_25-35_ vs. COT + Aβ_25-35_: *p* < 0.0001; (**B**) * Control vs. NIC + Aβ_25-35_, Aβ_25-35_ vs. 6HLN + Aβ_25-35_, 6HLN + Aβ_25-35_ vs. COT + Aβ_25-35_: *p* < 0.01, ** Control vs. Aβ_25-35_: *p* < 0.001, *** NIC vs. Aβ_25-35_, 6HLN vs. Aβ_25-35_, COT vs. Aβ_25-35,_ Aβ_25-35_ vs. NIC + Aβ_25-35_, Aβ_25-35_ vs. COT + Aβ_25-35_: *p* < 0.0001; (**C**) *** Control vs. Aβ_25-35_, NIC vs. Aβ_25-35_, 6HLN vs. Aβ_25-35_, COT vs. Aβ_25-35,_ Aβ_25-35_ vs. NIC + Aβ_25-35_: *p* < 0.0001, * Aβ_25-35_ vs. 6HLN + Aβ_25-35_, Aβ_25-35_ vs. COT + Aβ_25-35_, NIC + Aβ_25-35_ vs. 6HLN+ Aβ_25-35_, NIC + Aβ_25-35_ vs. COT + Aβ_25-35_: *p* < 0.01.

Figure 9A indicates that brain infusion of Aβ_25-35_ peptide induces a significant decrease (*p* < 0.001) of *bdnf* mRNA copy number compared to the control group, suggesting a neurotoxic effect of the peptide. However, this parameter was reversed by the chronic pre-treatment (0.3 mg/kg, b.w., i.p.) with COT and 6HLN, which significantly increased (*p* < 0.0001) the *bdnf* mRNA copy number compared to the group treated with peptide alone. These data showed that COT and 6HLN increase the *bdnf* gene expression, thus suggesting a neuroprotective effect against Aβ_25-35_ peptide-induced neurotoxicity.

In agreement with previous studies, our results suggest that COT and 6HLN could possess neuroprotective properties by increasing the *bdnf* gene expression in animal models of dementia.

In vitro studies indicated that COT exhibited neuroprotective activity on neuronal cells against Aβ [94,115] and 6-OHDA [116] neurotoxicity.

#### 3.5.2. Effects of Nicotinic Derivatives on Arc Expression

Activity-regulated cytoskeletal-associated protein, encoded by the immediate early gene *arc* (*arg3.1*), is a neuron-specific and post-synaptic protein that is essential in synaptic plasticity and consolidation of explicit and implicit forms of memory [117,118]. In AD, synaptic dysfunction was highly correlated with levels of soluble Aβ oligomers, and it was demonstrated that these oligomers are targeting synapses and alter the spines’ morphology and density. These synaptic alterations were correlated with memory deficits that occurred in AD [118,119]. Interestingly, high levels of *arc* expression were reported in AD patients. However, several mouse models of AD exhibited a low number of Arc-expressing cells [120], a reduced *arc* mRNA expression following explorative behavior [120,121] and lower levels of *arc* mRNA in the brain regions that contain Aβ [122]. To evaluate the role of nicotinic derivatives on memory consolidation, we examined the *arc* mRNA copy number in the rat hippocampus after performing the memory tasks.

Figure 9B shows that the brain delivery of Aβ_25-35_ peptide significantly reduced (*p* < 0.001) the *arc* mRNA copy number in rat hippocampus compared to the sham-operated control group, suggesting an abrogation of memory consolidation that could also affect the protein synthesis required to produce synaptic changes needed for long-term memory storage. However, chronic treatment (0.3 mg/kg, b.w., i.p.) with 6HLN and COT significantly increased (*p* < 0.01 for 6HLN and *p* < 0.0001 for COT) the *arc* mRNA copy number in the hippocampus of the Aβ_25-35_-treated rats (Figure 9A). Of the two nicotinic derivatives, COT was found to be more effective than 6HLN in increasing the *arc* mRNA expression (*p* < 0.01). Therefore, these data indicate that COT and 6HLN improve the memory consolidation process in Aβ_25-35_-treated rats by increasing the *arc* mRNA level in the hippocampus.

In accordance with previous findings, our results indicate that 6HLN and COT increase the *arc* gene expression in rat hippocampus, improving the memory consolidation process that was impaired by Aβ_25-35_ peptide. Kristensen et al. [123] showed that the α7 nAChRs could have an important role in *arc* expression, as a dose- and time-dependent increase in *arc* mRNA level was observed in the prefrontal cortex of the rats treated with a selective α7 receptor partial agonist. The α7 nAChRs modulation by COT could stimulate CREB and ERK1/2, which might promote the Arc expression [23,124]. Moreover, it has been shown that BDNF stimulates Arc expression and is able to directly activating Arc-dependent long-term potentiation (LTP) consolidation [125]. This might suggest that the increase in *arc* gene expression observed in this study could also be attributed to the enhancing effect of COT and 6HLN on the *bdnf* expression discussed above. These results indicate that COT and 6HLN might sustain the memory consolidation process by upregulating *arc* gene expression after nAChRs modulation.

#### 3.5.3. Effects of Nicotinic Derivatives on il-1β Expression

Interleukin-1β (IL-1β) is an essential proinflammatory cytokine involved in the regulation of the host’s innate immune response [126]. Clinical and preclinical studies demonstrated that IL-1β plays a central role in inducing pathogenic neuroinflammation of AD [127,128]. In AD-affected elevated levels of IL-1β have been reported. In a transgenic mouse model of AD (APP_Swe_/PS1ΔE9), an increase in IL-1β expression was observed in microglia that clustered around amyloid plaques [128]. In addition, the mice lacking the IL-1 receptor antagonist, an endogenous blocker of IL-1 receptor, showed an increased microglial activation and neuronal damage after i.c.v. infusion of Aβ [129]. IL-1β has also been shown to increase APP expression and Aβ production in astrocytes [130] and can cause protein tau phosphorylation via MAPK-p38 pathway to form NFTs [131]. In the current study, we evaluated the effects of nicotinic derivatives on neuroinflammation by examining the *il-1β* gene expression in Aβ_25-35_-treated rat’s hippocampus.

Figure 9C reports the effects of Aβ_25-35_ peptide infusion along with COT and 6HLN treatments (0.3 mg/kg, b.w., i.p.) on *il-1β* mRNA copy number. Aβ_25-35_ peptide infusion significantly elevated (*p* < 0.0001) the *il-1β* mRNA copy number compared to the sham-operated control group, suggesting an inflammatory process in the rat’s hippocampus. However, chronic administration of COT and 6HLN significantly reduced (*p* < 0.01) the *il-1β* mRNA copy number in the brain of Aβ_25-35_-treated rats compared with the group treated with the peptide alone, indicating an anti-inflammatory potential of the treatment.

Our results indicate that COT and 6HLN might reduce the Aβ_25-35_-induced neuroinflammation by decreasing the *il-1β* gene expression. A previous study performed on primary human monocytes demonstrated that the pre-treatment with COT for 2 h blocked the inflammatory response induced by Gram-positive bacteria by suppressing the cytokines production that is under the transcriptional control of the NF-κB system, such as TNF-α, IL-1β, IL-6, IL-12/IL-23 and p40, and by augmenting the IL-10 production [132]. COT blocked more than 80% of *Porphyromonas gingivalis*-induced TNF-α release. This effect was counteracted by α-bungarotoxin, thus suggesting that this anti-inflammatory effect of COT was mediated by α7 nAChRs, and NF-κB-independent [132]. Moreover, it was noticed that the effect is dependent on phosphatidylinositol 3 kinase (PI3K) and is accompanied by Akt activation and GSK3β inhibition by Ser9 phosphorylation (Akt phosphorylation site) [132]. A similar Akt activation and GSK3β inhibition were also noticed in brain homogenates of Tg6799 mice treated with COT [30]. The anti-inflammatory effects of COT involved Toll-like receptors (TLRs) activation. In fact, in monocytes, COT blocked cytokine production, resulting in TLRs-activation (TLRs 2/1, 2/6, 4, and 5) by a specific antagonist [133]. Here, we have shown for the first time that 6HLN might reduce the Aβ_25-35_-induced neuroinflammation in the rat hippocampus by normalizing the *il-1β* gene expression. Taken together, COT and 6HLN might possess anti-inflammatory properties following nAChRs-modulation.

## 4. Conclusions

By using in silico tools, we predicted that COT and 6HLN could bind and modulate the α7 and α4β2 subtypes of nAChRs. In the α4β2 nAChRs, the ligands exhibited a stronger affinity towards α4-β2 site compared to the α4-α4 site. Moreover, this study demonstrated that COT and 6HLN were able to ameliorate Aβ_25-35_-induced cognitive deficits. In addition, the chronic treatment with nicotinic derivatives also reduced the brain oxidative stress and AChE activity, suggesting an antioxidant and anti-cholinesterase potential of COT and 6HLN. Finally, the RT-qPCR analysis revealed that COT and 6HLN might possess neuroprotective, proamnesic, and anti-inflammatory properties by positively modulating the expression of *bdnf*, *arc* and *il-1β* genes, respectively. These effects were attributed to the nAChRs-modulation, and this strengthens the idea that these compounds might represent viable therapeutic agents for AD.

## Figures and Tables

**Table 1:** The receptors and the binding sites used in docking experiments.

Receptor	Site Name	Site Description	Resolution (Å)	PDB Entry	Reference
AChBP	AChBP	The interface between two identical subunits	2.2	1UW6	[32]
α4β2 nAChR (3α:2β)	α4-α4	The interface between two α4 subunits	3.9	6CNK	[33]
	α4-β2	The interface between an α4 and β2 subunit			

**Table 2:** Primers used to amplify targeted genes.

Gene	Product Size (bp)	Primer	Sequence
*bdnf* (exon 5)	101	Forward	5′-ATT ACC TGG ATG CCG CAA AC-3′
		Reverse	5′-TGA CCC ACT CGC TAA TAC TGT-3′
*arc*	115	Forward	5′-CCCTGCAGCCCAAGTTCAAG-3
		Reverse	5′-GAAGGCTCAGCTGCCTGCTC-3′
*il-1β*	144	Forward	5′-AGC ACC TTC TTT TCC TTC ATC TT-3′
		Reverse	5′-CAG ACA GCA GGC ATT TT-3′

**Table 3:** The root mean square deviation (RMSD), binding energies and ligand efficiencies for the best three binding poses of the ligands in the corresponding receptor.

Ligands	Binding Poses *	Receptor (Binding Site)								
		AChBP			α4-α4			α4-β2		
		RMSD	Binding Energy	Ligand Efficiency	RMSD	Binding Energy	Ligand Efficiency	RMSD	Binding Energy	Ligand Efficiency
(*S*)-Nicotine	1	0.46	−7.05	−0.59	0.55	−5.57	−0.46	0.47	−6.06	−0.51
	2	0.45	−6.95	−0.58	0.56	−5.51	−0.46	0.31	−5.93	−0.49
	3	0.29	−6.59	−0.55	0.23	−5.5	−0.46	0.32	−5.93	−0.49
(*R*)-Nicotine	1	0.36	−6.88	−0.57	0.5	−5.52	−0.46	0.48	−5.87	−0.49
	2	0.37	−6.79	−0.57	0.51	−5.51	−0.46	0.62	−5.86	−0.49
	3	0.57	−6.77	−0.56	0.51	−5.49	−0.46	0.47	−5.85	−0.49
(*S*)-Cotinine	1	0.27	−6.6	−0.51	0.4	−5.65	−0.43	0.45	−6.24	−0.48
	2	0.5	−6.56	−0.5	0.45	−5.62	−0.43	0.61	−6.17	−0.47
	3	0.56	−6.46	−0.5	0.47	−5.61	−0.43	0.45	−6	−0.46
(*R*)-Cotinine	1	0.6	−6.55	−0.5	0.45	−5.65	−0.43	0.45	−6.17	−0.47
	2	0.44	−6.47	−0.5	0.54	−5.62	−0.43	0.44	−6.17	−0.47
	3	0.42	−6.42	−0.49	0.43	−5.62	−0.43	0.45	−6.13	−0.47
(*S*)-6-Hydroxynicotine	1	0.33	−7.2	−0.55	0.28	−5.71	−0.44	0.18	−6.16	−0.47
	2	0.27	−7.18	−0.55	0.37	−5.69	−0.44	0.22	−6.16	−0.47
	3	0.25	−7.17	−0.55	0.39	−5.67	−0.44	0.18	−6.16	−0.47
(*R*)-6-Hydroxynicotine	1	0.64	−7.34	−0.56	0.42	−5.73	−0.44	0.51	−6.48	−0.5
	2	0.5	−7.34	−0.56	0.65	−5.69	−0.44	0.6	−6.37	−0.49
	3	0.65	−7.32	−0.56	0.51	−5.66	−0.44	0.5	−6.36	−0.49

* The best three binding poses of the ligands selected based on RMSD, binding energy and ligand efficiency.

## Supplementary Materials

The following are available online at https://www.mdpi.com/2076-3921/9/8/768/s1 Figure S1: The native position of the nicotine molecule at the interface between two identical subunits of AChBP (1UW6) (**A**) and the best theoretical binding positions of (S)-nicotine (**B**), (S)-cotinine (**C**) and (S)-6-Hydroxynicotine (**D**). The ligands are displayed as ball and sticks, the NIC binding site-residues are shown as sticks–the residues with side chain colored in magenta belong to the principal side (+) while the residues with side chain in dark blue belongs to the complementary side (-), the hydrogen bonds are represented as green dashed lines and the rest of the receptor as surface, Figure S2: Schematic diagram of the interactions between the residues of the nicotine-binding interface of AChBP and nicotine (**A**), (S)-nicotine (**B**), (S)-cotinine (**C**) and (S)-6-hydroxynicotine (**D**). The residues that are marked with * are common with those from literature and the encircled residues are common for all the selected ligands. The hydrophobic contact(s) between the residues and the corresponding atoms of the ligands as well as the hydrogen bonds are illustrated according to the Ligplot+ software, Figure S3: The native position of the nicotine molecule in the α4-α4 site of α4β2 nAChRs (3α:2β, 6CNK) (**A**) and the best theoretical binding positions of (S)-nicotine (**B**), (S)-cotinine (**C**) and (S)-6-Hydroxynicotine (**D**). The ligands are displayed as ball and sticks, the NIC bindingsite-residues are shown as sticks, the residues with side chain colored in magenta belong to the principal side α4+ while the residues with side chain in dark blue belongs to the complementary side α4-, the hydrogen bonds are represented as green dashed lines and the rest of the receptor as surface, Figure S4: Schematic diagram of the interactions between the residues of α4-α4 nicotine-binding interface of α4β2 nAChRs and nicotine (**A**), (S)-nicotine (**B**), (S)-cotinine (**C**) and (S)-6-hydroxynicotine (**D**). The residues that are marked with * are common with those from literature and the encircled residues are common for all the selected ligands. The hydrophobic contact(s) between the residues and the corresponding atoms of the ligands as well as the hydrogen bonds are illustrated according to the Ligplot+ software, Figure S5: Schematic diagram of the interactions between the residues of α4-β2 nicotine-binding interface of α4β2 nAChRs and nicotine (**A**), (S)-nicotine (**B**), (S)-cotinine (**C**) and (S)-6-hydroxynicotine (**D**). The residues that are marked with * are common with those from literature and the encircled residues are common for all the selected ligands. The hydrophobic contact(s) between the residues and the corresponding atoms of the ligands as well as the hydrogen bonds are illustrated according to the Ligplot+ software.
