# Supplementary material for: Cotinine and 6-Hydroxy-L-Nicotine Reverses Memory Deficits and Reduces Oxidative Stress in Aβ25-35-Induced Rat Model of Alzheimer’s Disease
Source: Antioxidants (Basel). 2020 Aug 18;9(8):768. doi: 10.3390/antiox9080768 (PMC7465470; doi:10.3390/antiox9080768)
Supplement: Supplementary file 1 [file antioxidants-09-00768-s001.pdf]

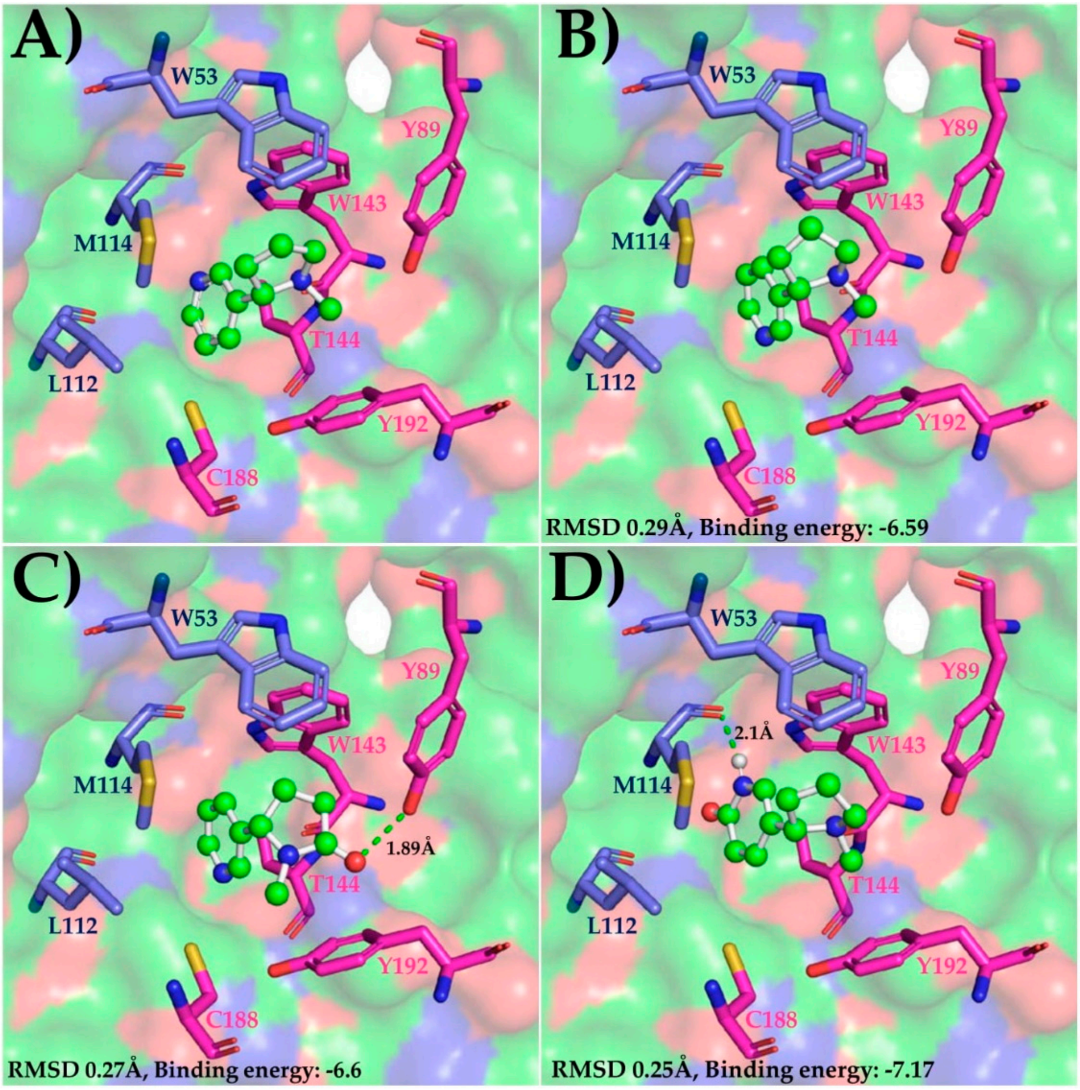

Figure 1. The native position of the nicotine molecule at the interface between two identical subunits of AChBP (1UW6) (**A**) and the best theoretical binding positions of (S)-nicotine (**B**), (S)-cotinine (**C**) and (S)-6-Hydroxynicotine (**D**). The ligands are displayed as ball and sticks, the NIC binding site-residues are shown as sticks – the residues with side chain colored in magenta belong to the principal side (+) while the residues with side chain in dark blue belongs to the complementary side (-), the hydrogen bonds are represented as green dashed lines and the rest of the receptor as surface.

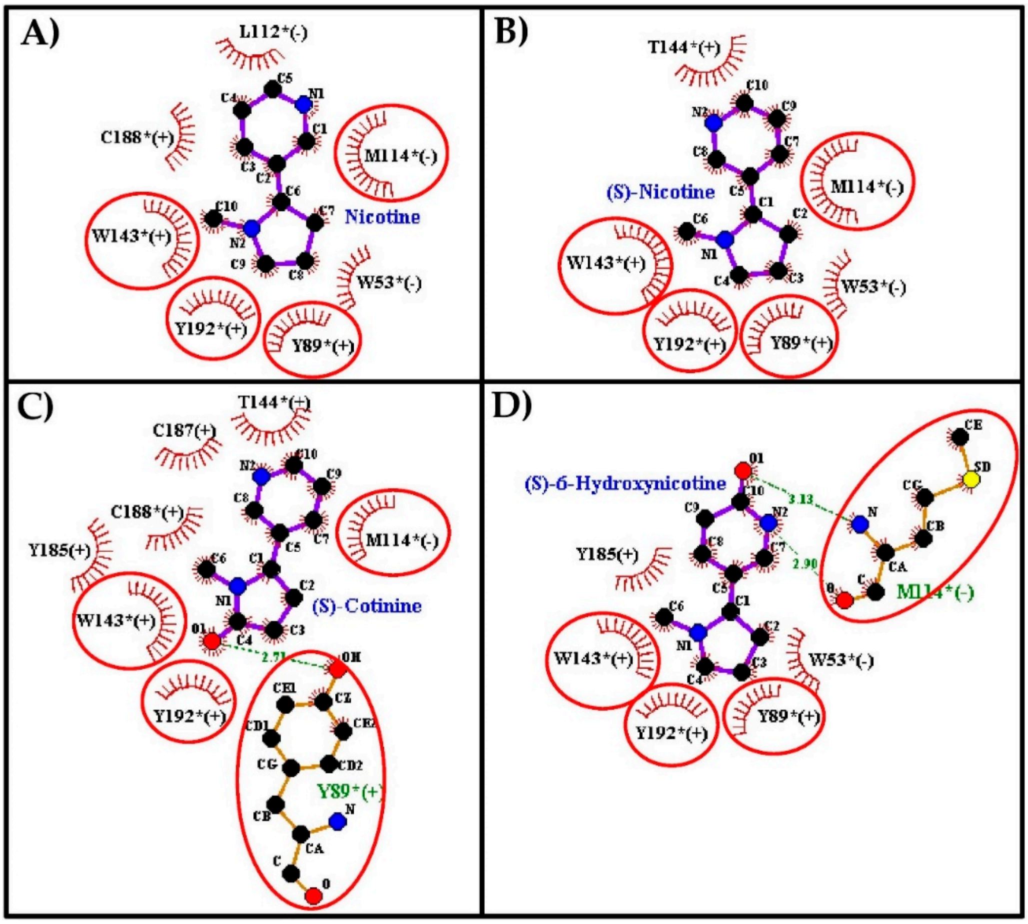

Figure 2. Schematic diagram of the interactions between the residues of the nicotine-binding interface of AChBP and nicotine (**A**), (S)-nicotine (**B**), (S)-cotinine (**C**) and (S)-6-hydroxynicotine (**D**). The residues that are marked with * are common with those from literature and the encircled residues are common for all the selected ligands. The hydrophobic contact(s) between the residues and the corresponding atoms of the ligands as well as the hydrogen bonds are illustrated according to the Ligplot+ software.

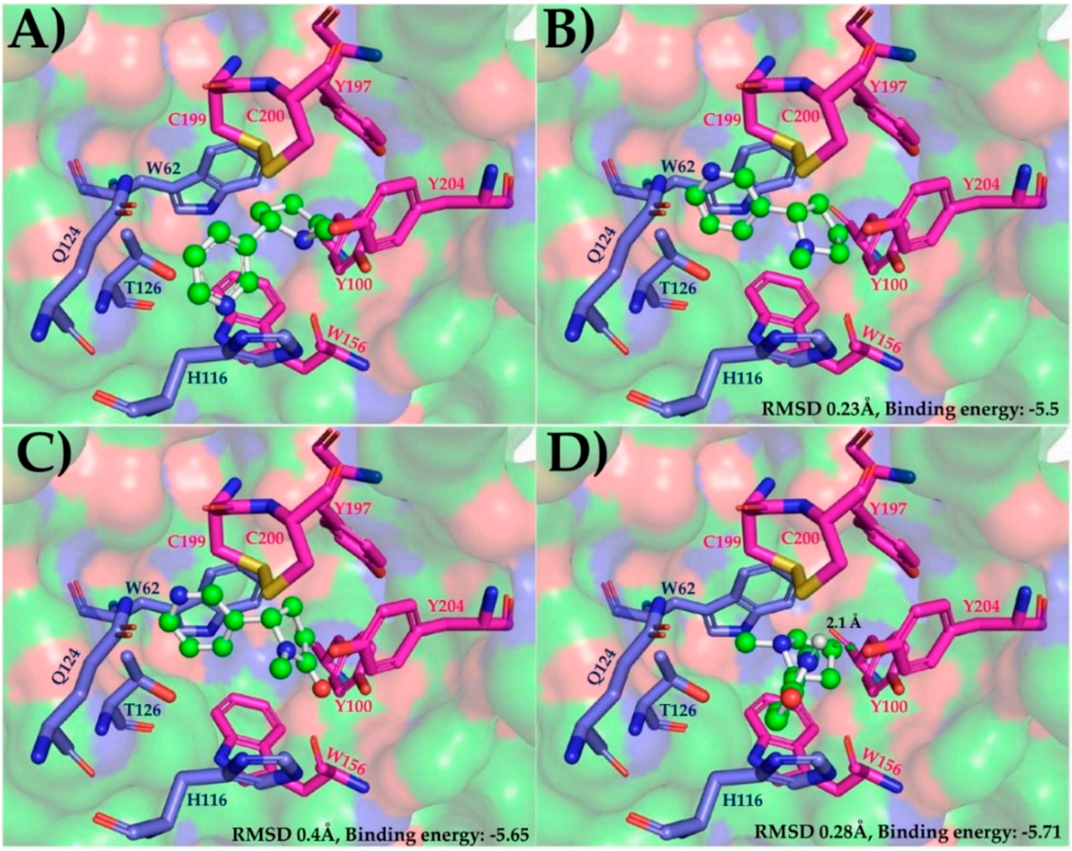

Figure 3. The native position of the nicotine molecule in the α4-α4 site of α4β2 nAChRs (3α:2β, 6CNK) (**A**) and the best theoretical binding positions of (S)-nicotine (**B**), (S)-cotinine (**C**) and (S)-6-Hydroxynicotine (**D**). The ligands are displayed as ball and sticks, the NIC bindingsite-residues are shown as sticks, the residues with side chain colored in magenta belong to the principal side α4+ while the residues with side chain in dark blue belongs to the complementary side α4-, the hydrogen bonds are represented as green dashed lines and the rest of the receptor as surface.

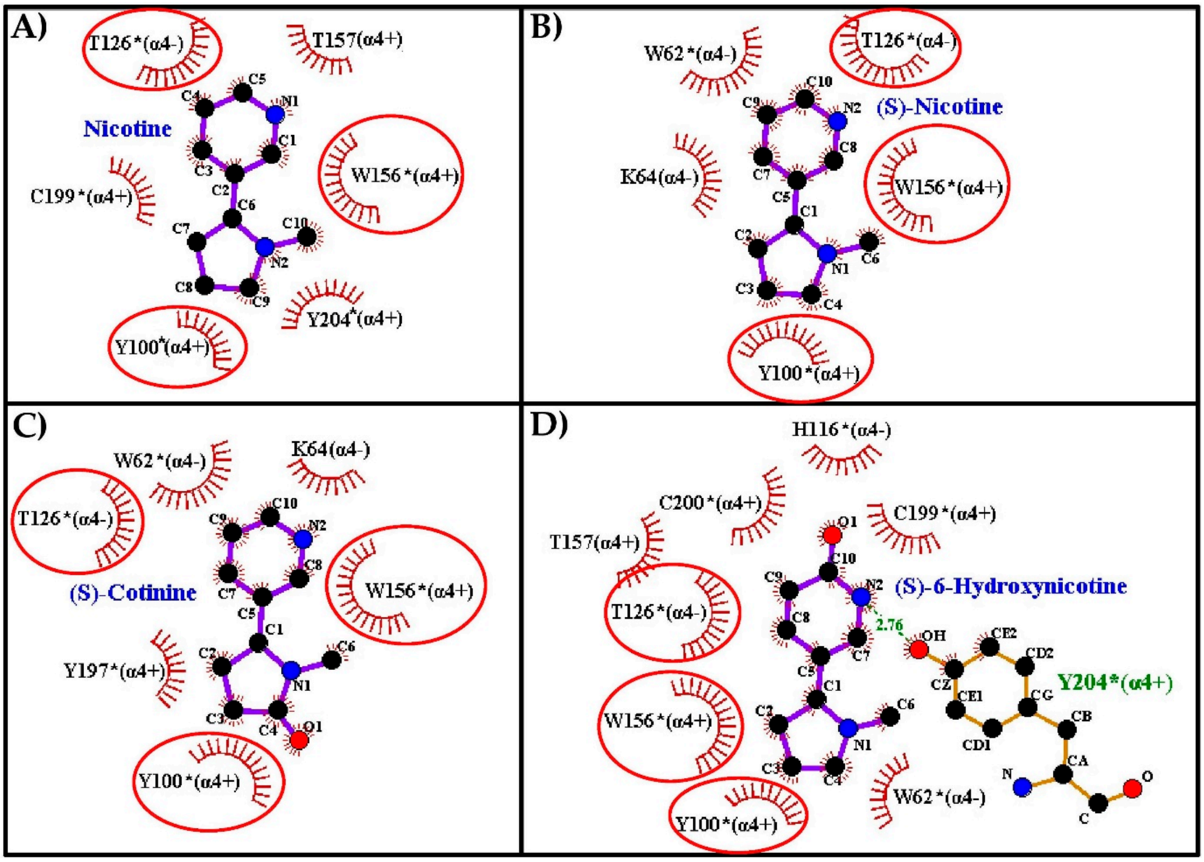

Figure 4. Schematic diagram of the interactions between the residues of α4-α4 nicotine-binding interface of $\alpha4\beta2$ nAChRs and nicotine (**A**), (*S*)-nicotine (**B**), (*S*)-cotinine (**C**) and (*S*)-6-hydroxynicotine (**D**). The residues that are marked with * are common with those from literature and the encircled residues are common for all the selected ligands. The hydrophobic contact(s) between the residues and the corresponding atoms of the ligands as well as the hydrogen bonds are illustrated according to the Ligplot+ software.

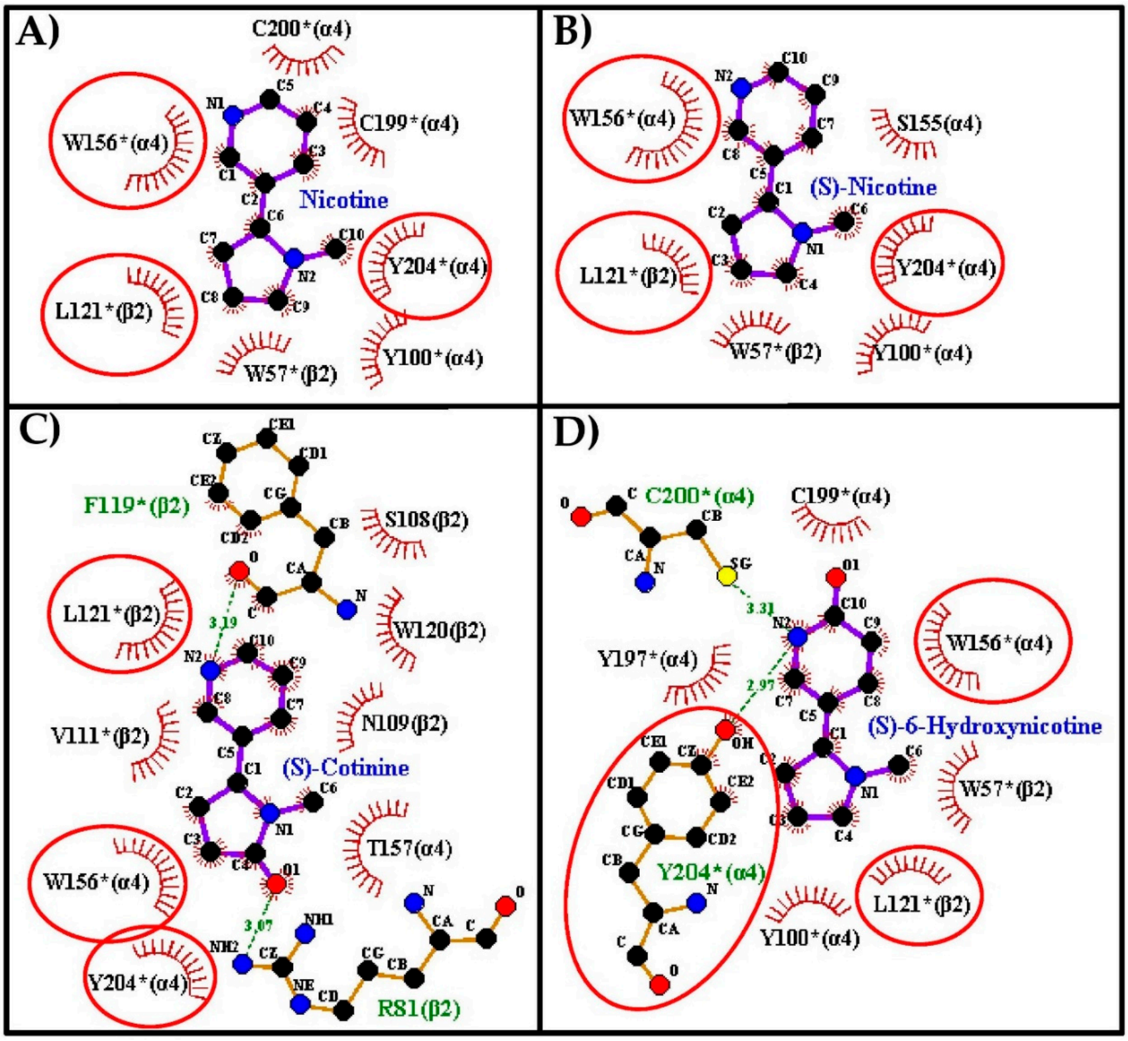

Figure 5. Schematic diagram of the interactions between the residues of *α*4-β2 nicotine-binding interface of *α*4β2 nAChRs and nicotine (**A**), (*S*)-nicotine (**B**), (*S*)-cotinine (**C**) and (*S*)-6-hydroxynicotine (**D**). The residues that are marked with * are common with those from literature and the encircled residues are common for all the selected ligands. The hydrophobic contact(s) between the residues and the corresponding atoms of the ligands as well as the hydrogen bonds are illustrated according to the Ligplot+ software.
